# Measuring human capital with social media data and machine learning

**DOI:** 10.1038/s41598-026-53076-4

**Published:** 2026-06-04

**Authors:** Martina Jakob, Sebastian Heinrich

**Affiliations:** 1 Department of Economics, University of Zurich, Zurich, Switzerland; 2 Department of Social Sciences, University of Bern, Bern, Switzerland; 3 Department of Management, Technology, and Economics, ETH Zurich, Zurich, Switzerland

**Keywords:** Machine learning, Social media data, Education, Human capital, Indicators, Natural language processing, Mathematics and computing, Science, technology and society

## Abstract

Timely data on educational attainment at granular geographic levels remains scarce in many countries, limiting evidence-based policy-making. Recent advances in machine learning have enabled the use of non-traditional data sources like satellite imagery and mobile phone records to measure development indicators. While these approaches have been successful in predicting outcomes such as wealth, poverty, or population density, previous attempts to predict educational attainment have achieved only modest accuracy. Here we show that language patterns and user behavior in social media can explain up to 70 percent of the variance in regional educational attainment. Our machine learning framework leverages linguistic features, user behavior, and network characteristics from 25 million geolocated tweets from the United States and Mexico. It performs particularly well in predicting higher education levels and maintains a good performance even with limited data collection periods. These results show that digital communication patterns can serve as reliable proxies for human capital. In light of the rapid expansion of social media use around the globe, this represents a promising approach to tracking educational outcomes in regions lacking granular and timely survey data.

## Introduction

Reliable data on key socio-economic outcomes enables policy-makers to make informed decisions and promote societal development. However, many countries are plagued by a pervasive lack of such data, limiting their ability to track progress and evaluate policies. To address the problem, a growing body of literature uses alternative data sources such as satellite imagery or phone records to bridge the existing gaps in data availability^1^. While previous studies have successfully predicted outcomes such as wealth, income or population density, this paper proposes an innovative approach to measuring human capital using geolocated Twitter data (now *X*).

We focus on educational attainment as our measure of human capital^2,3^, and validate our approach with two countries that differ markedly in language, economic development, and social media penetration: the United States and Mexico. Specifically, we construct a set of interpretable measures of education at low administrative units (municipality in Mexico and county in the United States) based on over 25 million tweets. Our feature matrix includes basic Twitter penetration (e.g., user density) and usage statistics (e.g., tweet length), text-based indicators on spelling mistakes (e.g., frequency of grammatical errors), topics (e.g., share of tweets about science), and sentiments (e.g., share of negative tweets), as well as network indicators (e.g., closeness centrality). We train a stacking regressor combining five machine learning algorithms — elastic net regression, gradient boosting, support vector regression, nearest neighbor regression, and a feed-forward neural network — to predict educational attainment for Mexican municipalities (N = 2,457) and US counties (N = 3,141). We apply grid search to tune the relevant hyperparameters of each model, and evaluate the performance of the final models using five-fold cross-validation and leave-one-state-out (LOSO) cross-validation.

Our predictions account for 70 percent of the variation in years of schooling in Mexican municipalities and 65 percent in US counties. Where, how and what people tweet is thus highly informative about human capital. Within both countries, Twitter data appears to be particularly well-suited for distinguishing higher levels of education. For example, we achieve an $R^2$ of 0.70 in predicting county-level shares of US adults holding a bachelor’s degree, while the corresponding $R^2$ for the percentage with a high school degree is only 0.50. We observe a similar though less pronounced relationship for Mexico, with an $R^2$ of 0.69 for the share with post-basic education and 0.61 for the percentage completing primary education. Our approach also generalizes well to entirely new geographic areas: LOSO cross-validation, which withholds all observations from one state at a time, yields $R^2$ values of 0.57 and 0.59 for years of schooling in Mexico and the United States, respectively.

Our focus on a limited number of meaningful features also allows us to study which (groups of) features are most predictive of educational outcomes. In most models, the user density emerges as the single most important predictor of educational outcomes. Twitter penetration features are particularly informative in Mexico, where they alone account for 57 percent of the variation in educational outcomes, compared to 37 percent in the US. Similarly, error and network features appear to be strongly related to education in Mexico ($R^2$ = 0.55 and 0.51, respectively), but less so in the US ($R^2$ = 0.42 and 0.34, respectively). General tweet statistics and topics have consistently high predictive power in Mexico and the United States ($R^2$ between 0.5 and 0.6).

The main challenge to model performance arises in sparsely populated areas with low Twitter penetration. Accordingly, the population-weighted $R^2$ for years of schooling is 0.85 for Mexico and 0.70 for the US (compared to 0.70 and 0.65 in our unweighted base model). Similarly, restricting the evaluation sample to areas with at least ten users would increase performance to 0.74 in Mexico and 0.68 in the US. We also explore how model performance evolves depending on the data collection period, finding that we can achieve relatively high predictive power with just three days of tweet data, namely an $R^2$ of 0.66 for Mexico and 0.58 for the United States.

Using wealth data for Mexico and income data for the US, we further explore how our measure of human capital performs in downstream tasks by comparing regression results based on predicted vs. ground truth measures of education. We find that slope coefficients tend to be biased not only when using the predicted indicator as an independent variable, but also when it acts as the dependent variable. The latter bias results from the typical model tendency to overpredict for low values and underpredict for high values. When using a loss function that penalizes quintile-specific biases (see Ratledge et al.^4^), the bias disappears, and regression coefficients based on our predicted indicator become very similar to their ground truth counterparts. Our simulations show that when appropriately modeled, predicted indicators can produce correct estimates in downstream regression tasks as long as they serve as the outcome and not the treatment variable.

This paper contributes to the recent literature exploring the combined potential of non-conventional data sources and machine learning to measure and understand socio-economic development. While a range of outcomes including wealth^5–8^, population density^9,10^, crop yield^11–13^, informal settlements^14,15^, electricity access^4^, and disease spread^16,17^ have been accurately predicted using satellite or phone data, previous attempts to infer human capital have been less successful. Head et al.^18^ use satellite data to predict educational attainment in Rwanda, Nigeria, Haiti and Nepal, achieving an average $R^2$ of *∼*0.55. The predictive power of other data sources, such as Google Street View images^19^ or Wikipedia articles^20^, appears to be even lower, accounting for less than 40 percent of the variation in educational outcomes. We show that by using geolocated Twitter data and natural language processing, we cannot only derive a more accurate indicator of human capital than previous studies but also achieve similar performance to the well-known wealth prediction using satellite data.

We also add to the literature leveraging social media data for social science research. Nearly five billion people worldwide used at least one social media platform in 2023, and another billion are projected to join by 2027, as emerging and developing economies catch up^21,22^. Social media data has been used to predict or study diverse outcomes such as migration^23,24^, social capital^25^, censorship^26^, alcohol consumption^27^ or stock market prices^28^. Moreover, micro-evidence suggests that social media posts are informative about educational characteristics of individual users^29,30^. This paper goes a step further and shows that despite the high endogenous selection in social media usage^31^, the corresponding data can be used to derive accurate education estimates at low administrative units within countries. This underscores the potential of social media data as part of a global tracking system that provides timely and granular information on educational outcomes.

## Data and methods

**Fig. 1:**
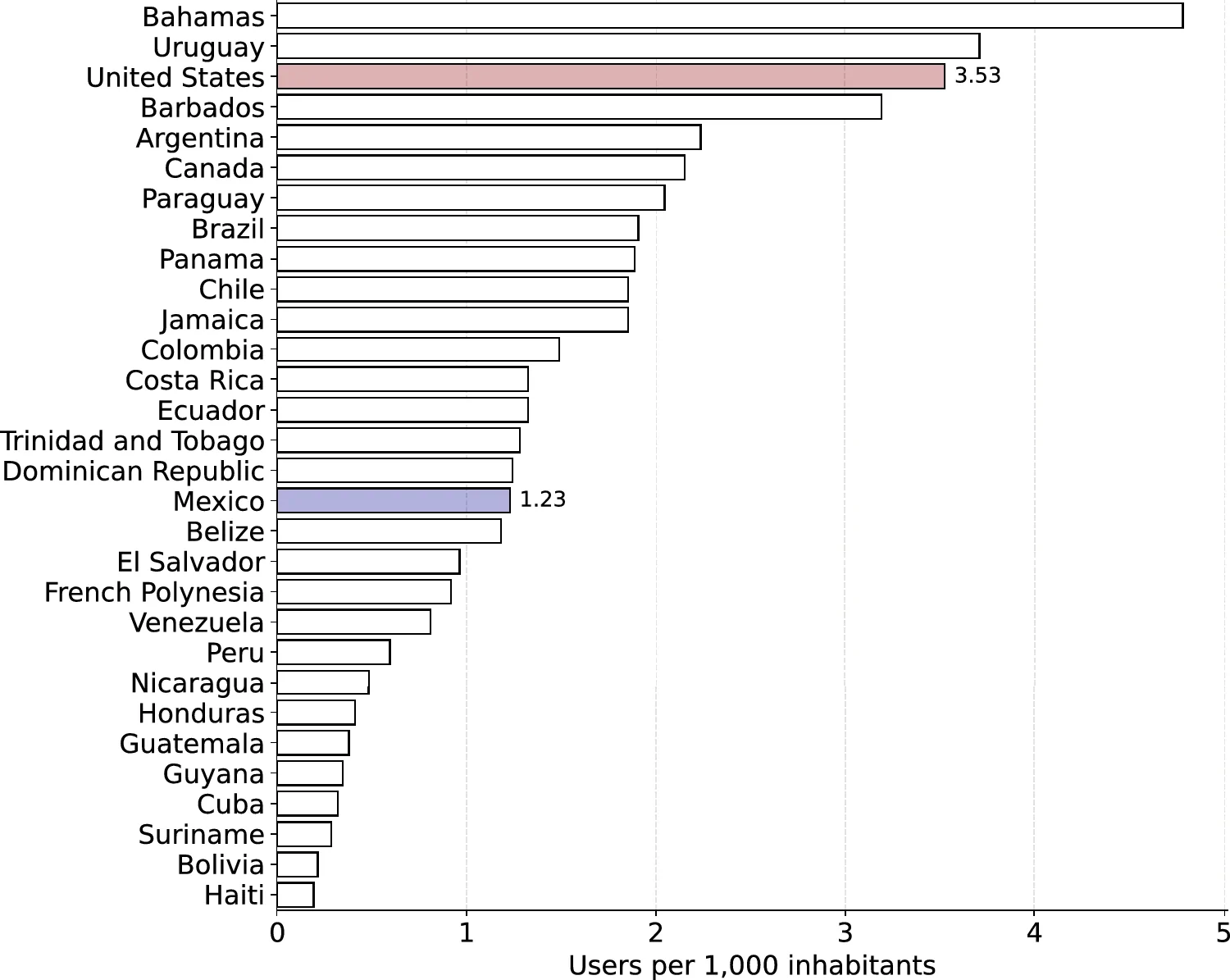
Twitter penetration across the Americas. Geolocated Twitter users per 1,000 inhabitants captured through the Twitter Streaming API in July-August 2021 for countries in North, Central, and South America.

To develop and validate our approach, we focus on two large countries with high-quality ground truth data on educational outcomes: the United States and Mexico. This pairing allows us to test our approach across different languages (English vs. Spanish), development contexts (high income vs. middle income), and, most importantly, levels of Twitter penetration (see Fig. 1). While the United States has one of the highest user densities in the Americas, Mexico ranks in the lower middle overall and has the lowest penetration among the five most populous countries in the region. By testing our approach in these diverse and data-rich contexts, we aim to establish a foundation for potential application in regions where traditional survey data are scarce or outdated.

### Collection and processing of Twitter data

We used the Twitter Streaming API to compile a large tweet dataset for both countries (see Appendix E for details on data collection and processing). Until early 2023, Twitter provided free access to real-time information on 1% of all tweets through its Streaming API, including the text of each tweet and a set of tweet and user characteristics. Our final dataset consists of 2,686,779 geolocated tweets from 123,309 users for Mexico and 22,610,134 tweets from 943,164 users for the United States, collected between July and August 2021.

The tweets included in our final dataset were selected based on three criteria:

1. *Geographical location*: We excluded all tweets that were not posted from within the geographic territory of the respective country. In the case of the United States, we use all tweets from the mainland, Alaska and Hawaii, but not from unincorporated territories such as Puerto Rico or the Virgin Islands. We also exclude tweets without precise location information (i.e., less precise than municipality/county level). Our final sample comprises tweets with exact coordinates (MX: 3%, US: 3%), neighborhood or point of interest (poi)-level precision coordinates (MX: 2%, US: 2%), and city-level precision coordinates (MX: 95%, US: 94%).
2. *Language*: For each country, only tweets written in the primary native language (i.e., Spanish for Mexico and English for the United States) are included.
3. *Source*: A major concern regarding the reliability of Twitter data is that many tweets are automatically disseminated through APIs rather than individually created by a human user. We thus restrict our sample to content that is posted through the four main channels for human users: iPhone, Android, iPad, and Instagram. This excludes tweets generated through third-party APIs from platforms such as Foursquare or CareerArc (approximately 1 percent of geolocated tweets in Mexico and 7 percent in the United States).

To compute municipality- or county-level statistics, we follow a three-step procedure. First, each tweet is assigned to a geographical unit (i.e., municipality or county) based on its coordinate data. While this is straightforward for exact coordinates, we apply different types of consistency checks to find the correct unit when coordinate information consists of a bounding box at the city, poi, or neighborhood level.

Next, we approximate the home municipality or county for each user. If users tweet from more than one geographical entity (MX: 33% of users, US: 35% of users), we assign all their tweets to the entity from which they tweeted the most. For users whose tweet counts are equally divided among two or more entities (MX: 1%, US: 2%), we use the number of tweets posted during non-work hours on weekdays as a tiebreaker. This procedure results in the reassignment of 14 percent of tweets in Mexico and 12 percent of tweets in the United States. Tweets that cannot be unambiguously assigned to a municipality through this procedure are dropped (MX: 0.4%, US: 0.2%).

**Fig. 2:**
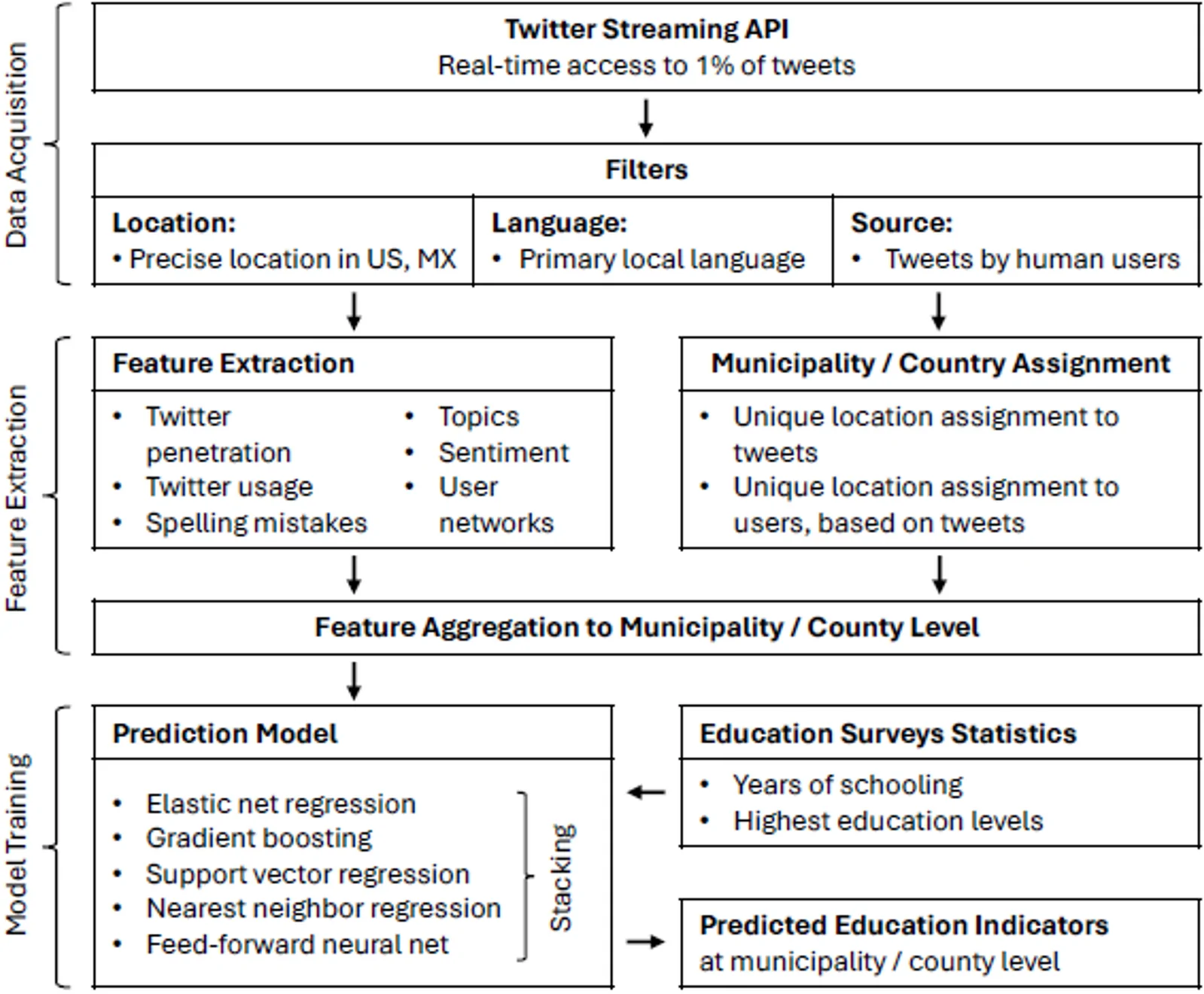
Data extraction and model training pipeline.

Finally, data is aggregated at the municipality or county level using the unit-level sum, mean, or median depending on the distribution of the underlying variables (for details, see Section “Features” and Appendix C). To give equal weight to all users irrespective of their degree of activity, all tweet-level variables are first aggregated at the user level (Fig. 2).

### Survey data

While many countries lack timely and spatially disaggregated information on educational outcomes, such data are available for both Mexico and the United States, allowing us to train and test a prediction algorithm in two different settings. Our main outcome variable is years of schooling for both countries, but we also look at the share of adults holding different educational degrees to better understand where in the educational distribution our models work best (see Table D.1). We use data from the 2020 census for Mexico and from the American Community Survey (2017–2021, 5-year estimates) for the United States, meaning that outcomes for both countries are temporally closely aligned with our input features from 2021. Following Barro and Lee^33^, we approximate county-level years of schooling for the US based on the proportion of the population holding different educational degrees and the average years of schooling associated with these degrees.

Section C in the Appendix presents summary statistics on all outcome variables. In the average Mexican municipality, 28 percent of the population holds a post-basic degree, 54 percent has graduated from secondary school, 76 percent has finished primary school, and the average person has completed 7.8 years of schooling. The corresponding figures in US counties are 23 percent with a bachelor degree, 54 percent with some college, 88 percent with a high school degree, and 13.3 years of schooling.

### Features

**Table 1:** Summary statistics by education level for selected features.

	Mexico			United States		
	Bottom 25%	Top 25%	All	Bottom 25%	Top 25%	All
User density	0.23	0.86	0.47	0.79	2.49	1.45
Tweet density	1.94	16.70	7.00	12.03	45.14	24.40
Tweet length	68.75	72.88	69.94	77.09	82.05	80.86
Account age	5.03	6.34	5.67	6.67	7.51	7.06
Tweets per year	1,306.55	362.71	841.93	648.93	351.58	495.19
Favorites per tweet	5.02	1.34	3.76	1.52	2.14	1.73
Error total	24.60	23.54	25.28	15.23	13.14	13.87
Error grammar	0.17	0.15	0.17	0.65	0.47	0.55
Error typos	12.18	10.66	12.47	7.48	6.92	7.19
Topic science	1.84	1.92	1.87	1.58	1.82	1.69
Topic relationships	6.66	5.72	6.27	5.31	4.42	4.76
Sentiment positive	0.39	0.37	0.38	0.50	0.50	0.50
Offensive language	0.15	0.16	0.15	0.17	0.16	0.16
Network clos. centr.	0.06	0.31	0.16	0.28	0.42	0.34
Number of Areas	430	429	1,714	723	723	2,889

Municipality (MX) or county (US) averages for selected features by educational outcome. The bottom 25% and top 25% refer to the municipalities/counties in the lowest or highest quartile of years of schooling. Only areas with at least one tweet are included. Features are not log transformed.

Our feature matrix comprises municipality-level information on *(i)* Twitter penetration, *(ii)* Twitter usage, *(iii)* spelling mistakes, *(iv)* topics, *(v)* sentiment, and *(vi)* user networks (for a detailed overview, see Section C and D in the Appendix). In addition, we include population density estimates, which also serve as our benchmark model against which the performance of our approach is compared. To advance our understanding of the aspects of people’s online behavior that are most predictive of human capital, we deliberately focus on a limited number of interpretable features rather than, for example, using tweet text embeddings. Importantly, the included features vary in how directly they capture education. In contrast to previous satellite-based approaches, several of our indicators are direct expressions of education (e.g., spelling mistakes, grammatical errors) or closely linked to educational attainment (e.g., different topics) rather than reflecting only the economic consequences of education. Nevertheless, other features such as Twitter penetration, usage patterns, and network characteristics are related to both education and broader socioeconomic conditions, meaning that our predictions partly capture regional socioeconomic outcomes correlated with education. The implications of this are discussed in detail in Section “Downstream performance”.

*Twitter penetration data* (4 features) consists of the total number of tweets and users as well as the number of users and tweets relative to the population (referred to as user and tweet densities). We further include general information on *Twitter usage* (11 features), such as the average tweet length, the number of followers, the user mobility, the account age, the number of emojis per tweet, or the share of tweets posted during work hours or from an iPhone. To obtain estimates for the frequencies of different *spelling mistakes* (MX: 23 features, US: 16 features), we use a Python wrapper for “LanguageTool”, an open source grammar, style, and spell checker. LanguageTool is available in over 25 languages, including English and Spanish, and classifies the detected errors into different categories such as grammar, typos, casing, punctuation, or style. We include the total number of errors per 1,000 characters and the corresponding numbers for each category. To determine the *topics* of each tweet (19 features), we use a pre-trained multi-label tweet classification model^34^. This allows us to estimate the probability a given tweet is about a particular topic, such as news, celebrity, sports, or science. Since no pre-trained tweet classification models are available in Spanish, we translate all Spanish tweets into English using a pre-trained model based on the Marian NMT framework^35^ to determine the topic distributions of our Mexican tweets. A further group of inputs comprises features related to *sentiments* (4 features), such as the share of tweets with negative or positive sentiments, offensive language, or hate speech. They are generated using pre-trained classification models for Spanish and English tweets. Finally, we also add *network indicators* (4 features), such as degree and closeness centrality. For this purpose, we use quotes and mentions to construct a user-to-user network and subsequently aggregate this network to the municipality or county level. We take the log of right-skewed features and standardize all features before training.

To address potential problems related to sparse or noisy data in areas of low population density, we develop a procedure that allows our model to learn from spatial neighbors. For each unit (i.e., municipality or county), we create a cluster consisting of the focal unit and all its spatial neighbors, and compute cluster-level estimates for each of our features. We use this information about Twitter usage in the broader area around each unit in three ways: First, we add the cluster-level estimates as additional inputs to our feature matrix (i.e., for each unit and measure, we include both unit- and cluster-level values). Second, we use cluster-level features to impute missing values in units without tweets using an elastic net regression model. This provides estimates for features that cannot be observed in the absence of tweets, and is necessary as most machine learning algorithms cannot handle missing values. Third, in units with fewer than 5 tweets, we replace extreme outliers with imputed values using the same imputation procedure. Extreme outliers are defined as values that are lower than $Q_{0.25} - 3 \, IQR$ or higher than $Q_{0.75} + 3 \, IQR$, with $Q_{0.25}$ and $Q_{0.75}$ as the first and third quartiles and *IQR* as the interquartile range.

Table 1 shows the mean of selected features by educational level for both countries (see Section C in the Appendix for complete summary statistics). This simple inspection already reveals a strong correlation between Twitter features and educational outcomes. In both countries, user and tweet density is markedly higher in places with more educated populations. Similarly, users in more educated areas tend to write longer tweets, make fewer errors, and talk about different topics (e.g., science rather than relationships). On the other hand, users in less educated areas tweet more actively.

### Training and evaluation

To train our models, we use a stacking regressor combining five machine learning algorithms: *(i)* elastic net regression, *(ii)* gradient boosting, *(iii)* support vector regression, *(iv)* nearest neighbor regression, and *(v)* a feed-forward neural network (i.e., a multi-layer perceptron). We use cross-validated grid search to tune the hyperparameters of each model. The performance of the final stacking regressor is evaluated using two complementary cross-validation approaches. First, we use *five-fold cross-validation*, where the data are randomly split into five folds and the model is trained on four folds and evaluated on the held-out fold in turn. This results in an overall procedure known as nested cross-validation. Second, we conduct *leave-one-state-out cross-validation *(e.g., Breen et al.^37^, Chi et al.^38^), where models are trained excluding all municipalities or counties from one state at a time and evaluated on the held-out state. This more stringent approach tests whether the model generalizes to entirely new geographic areas with potentially different socioeconomic contexts. For both approaches, we report the overall $R^2$ obtained by combining all out-of-sample predictions, where $R^2$ represents the coefficient of determination, i.e., $R^2 = 1 - \frac{\sum (y_i - \hat{y}_i)^2}{\sum (y_i - \bar{y})^2}$, where $y_i$ are observed values, $\hat{y}_i$ are out-of-sample predictions, and $\bar{y}$ is the mean of out-of-sample observations. All subanalyses, including robustness and generalizability checks, feature importance, heterogeneity analyses, and downstream performance assessments, are based on five-fold cross-validation.

## Results

### Main results

**Fig. 3:**
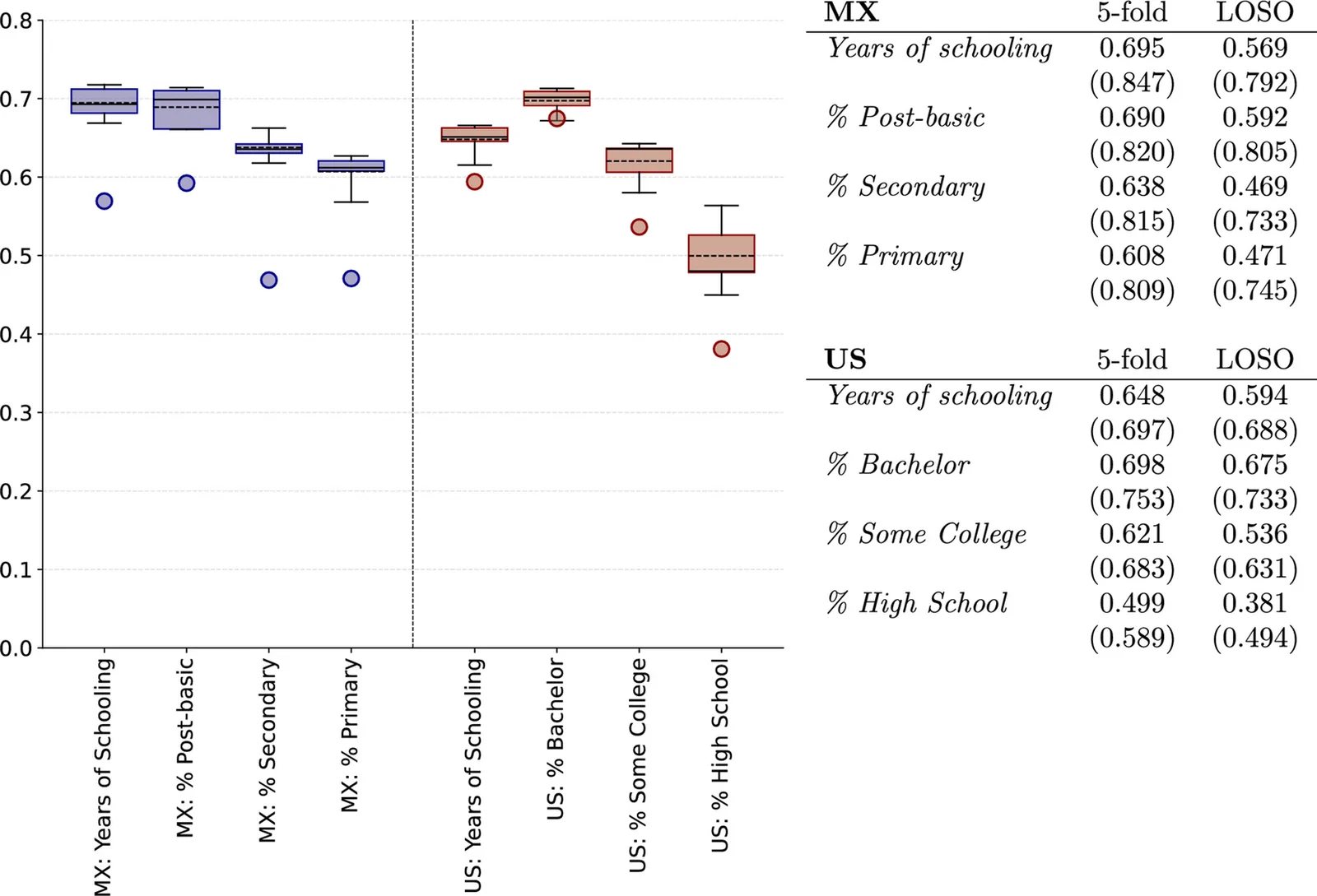
Performance for different educational outcomes. Model performance results for different educational outcomes in Mexican municipalities (blue) and US counties (red). Boxplots show the median (solid line), mean (dotted line), the 20th & 80th percentile (box limits), as well as the minimum & maximum (whiskers) for the $R^2$ across five-fold cross-validation folds. Circles indicate the leave-one-state-out (LOSO) cross-validation $R^2.$ The table presents the overall $R^2$ based on out-of-sample predictions combined across all folds (5-fold) or states (LOSO). Population-weighted $R^2$ are presented in parentheses.

When evaluated through *five-fold cross-validation*, our final model is able to account for 70 percent of the variation in years of schooling in Mexican municipalities and 65 percent in US counties (see Fig. 3). Population-weighted performance estimates are even higher, reaching an $R^2$ of 0.85 in Mexico and of 0.70 in the United States (Population weights are not taken into account during training). A closer look at the predictive power for different educational degrees reveals substantial variation in model performance in both countries.

In Mexico, we report an $R^2$ of 0.69 for the share of the population holding a post-basic degree (i.e., high school or more), an $R^2$ of 0.64 for the corresponding share with a secondary degree, and an $R^2$ of 0.61 when aiming to predict the prevalence of primary school completion. Differences are even more pronounced in the United States, where our model captures 70 percent of the variation in the percentage of adults that hold a bachelor’s degree, 62 percent for the share that went to college, and 50 percent when focusing on high school completion. This suggests that Twitter data is particularly informative about higher education levels and less sensitive to differences at the lower end of the education distribution. This pattern is likely driven by selection into platform usage, as more educated populations are more likely to use Twitter and generate sufficient data for reliable predictions.

**Fig. 4:**
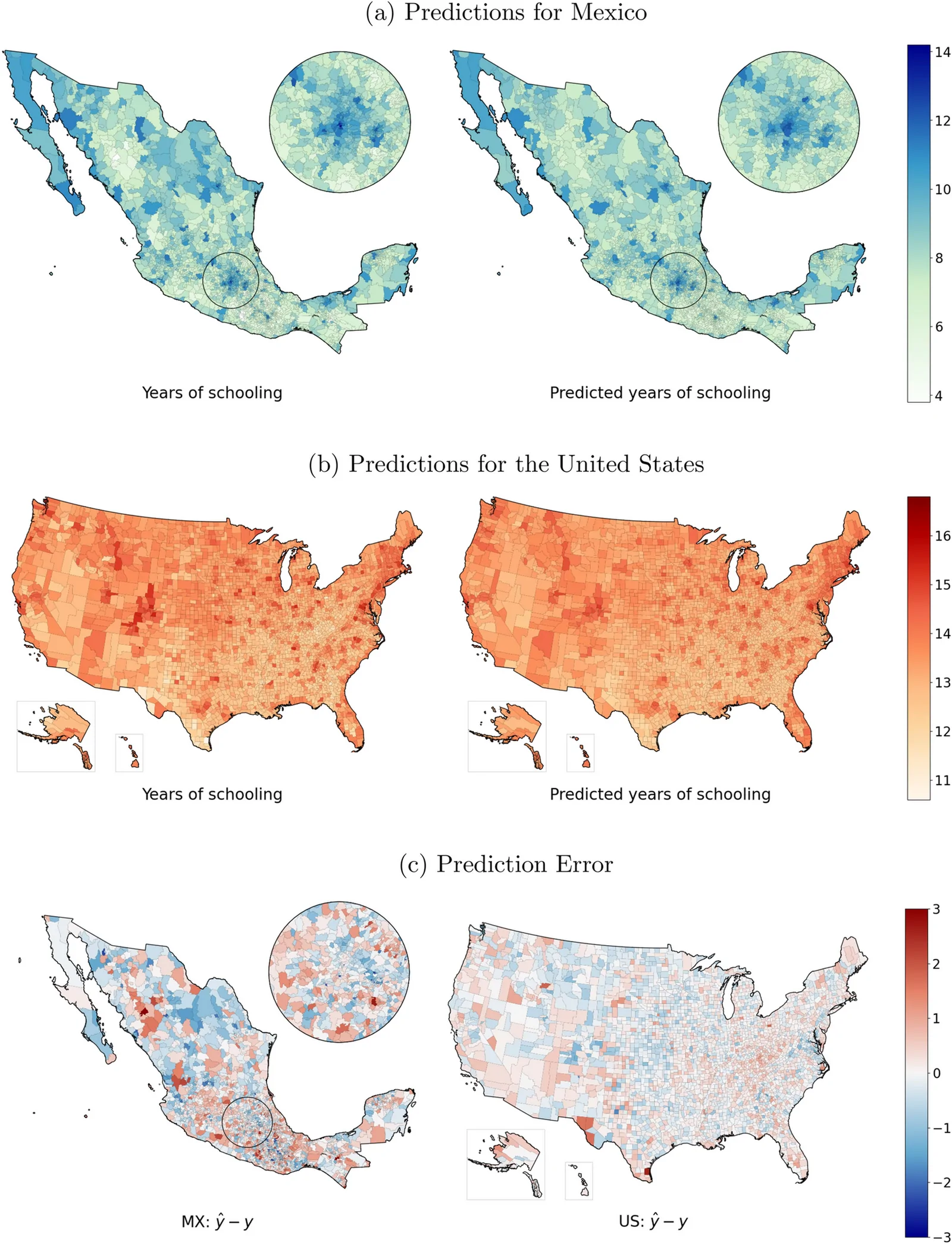
Maps of true vs. predicted years of schooling. Predicted values for all municipalities and counties are obtained by combining out-of-sample predictions from five-fold cross-validation. In Fig. 4c, red indicates overprediction and blue underprediction of true values.

**Fig. 5:**
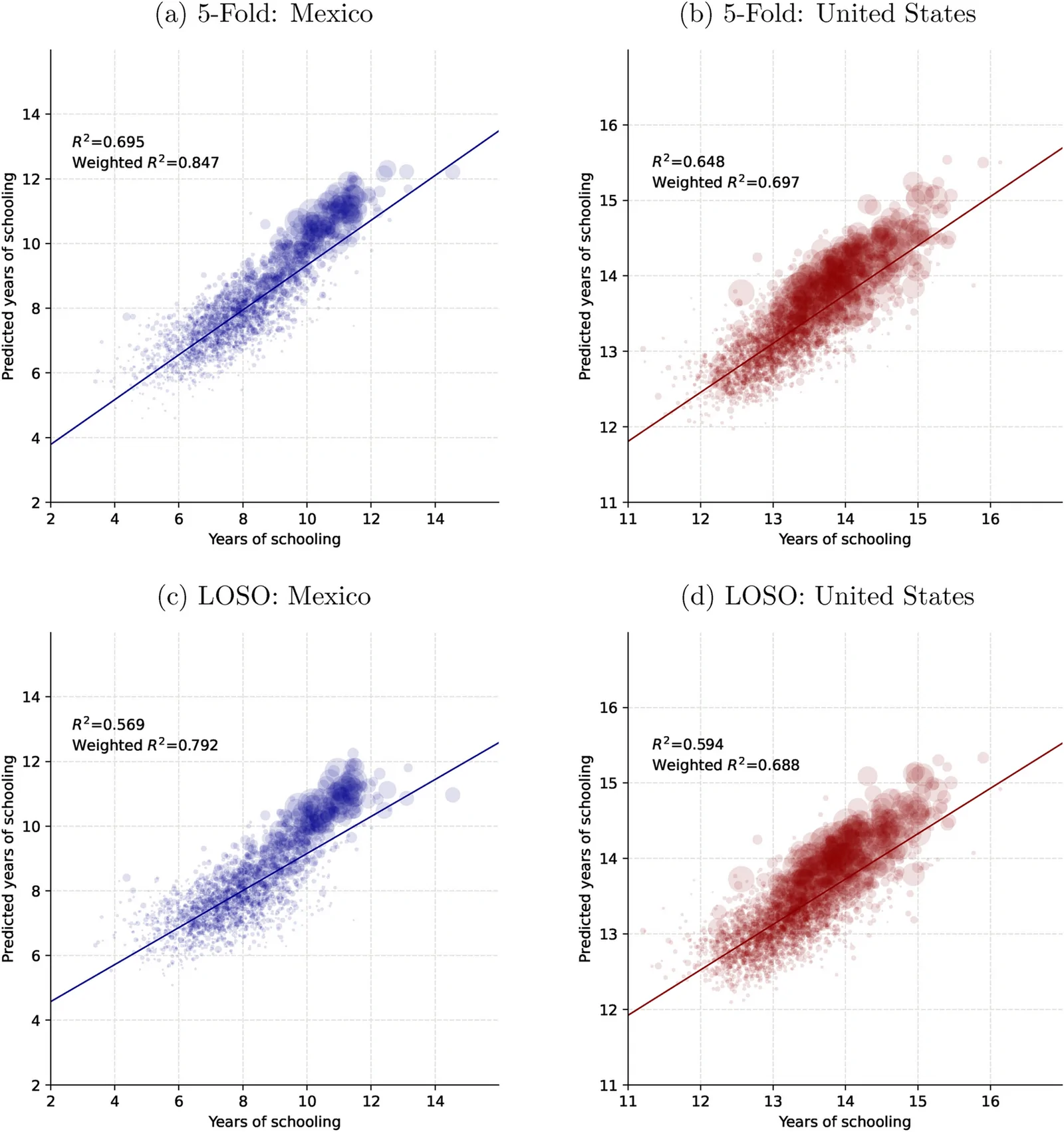
True vs. predicted years of schooling. Predicted values for all municipalities and counties are obtained by combining out-of-sample predictions across all folds (5-fold) or states (LOSO). Bubble size is proportional to the population in each unit. $R^2$ and population-weighted $R^2$ are shown. The line indicating the best linear fit is not population-weighted (many sparsely populated areas at the low end and few populous areas at the high end of the distribution create the illusion of a poor fit).

In line with previous research (e.g., Breen et al.,^37^, Chi et al.,^38^), the more stringent *leave-one-state-out cross-validation* (LOSO), which tests whether the model generalizes to entirely new geographic areas, yields lower performance estimates. For years of schooling, the unweighted $R^2$ is 0.57 in Mexico and 0.59 in the United States, and the population-weighted $R^2$ is 0.79 and 0.69, respectively. Results for other outcomes follow a similar pattern, with particularly pronounced performance differences in Mexico and for lower education levels. LOSO $R^2$ ranges from 0.47 to 0.59 (population-weighted: 0.73 to 0.81) in Mexico and from 0.38 to 0.68 (population-weighted: 0.49 to 0.73) in the United States (see Figs. 3, 5, and A.3). This corresponds to a decrease in $R^2$ of 2–17 percentage points in Mexico and 1–12 percentage points in the United States. Despite these reductions, performance remains strong for all but one outcome (% with a high school degree, US), highlighting the broader spatial transferability of our approach.

Among the five included models, gradient boosting and support vector regression perform best and, accordingly, receive the highest weights in the final stacking regressor (see Fig. A.1 and Table A.1 in the Appendix). The neural network and the nearest neighbor regressor, on the other hand, perform rather poorly, achieving a lower predictive power than the simple elastic net model (i.e., a regularized linear model). For all outcomes, the ensemble of all models outperforms the best-performing individual model, highlighting the benefits of stacking.

As Figs. 5a and b show, our model produces the attenuated predictions that are typical for continuous outcomes^4^, meaning that, on average, estimates are too high in low-education and too low in high-education areas. This pattern also becomes apparent when comparing maps of true and predicted years of schooling (see Figs. 4a and b). While spatial patterns look very similar for the two measures, they are slightly less fine-grained in the prediction maps.

### Robustness and generalizability

To assess the *reliability* of our predictions, we quantify uncertainty at two levels. First, the boxplots in Fig. 3 show variation in model performance across different cross-validation folds, indicating relatively stable performance with modest variation for most outcomes. Second, we assess prediction uncertainty at the unit level by re-running the full cross-validation procedure with different random fold assignments (see Appendix Section A.4 for details). We find that prediction uncertainty is generally higher in Mexican municipalities than in US counties, likely reflecting the larger underlying variance in years of schooling in Mexico. Within both countries, uncertainty is substantially higher in municipalities or counties with smaller populations, fewer Twitter users, and greater reliance on spatial imputation (Figs. A.7 and A.8). These patterns closely mirror those of prediction errors (Fig. A.9), and both uncertainty and errors exhibit spatial clustering (Fig. 4c and A.7).

We also assess the *temporal generalizability* of our approach. Given the substantial changes in Twitter’s data accessibility and user composition since our data collection period^39–41^, we examine whether our 2021 Twitter data remain predictive of more recent educational outcomes. For the United States, we use updated data from the most recent American Community Survey (2019–2023 compared to 2017–2021) to re-evaluate our models. Comparable updated data are not yet available for Mexico (most recent census: 2020, which we already use in our main analysis). Performance is very similar across all outcomes with declines of 0–2 percentage points compared to our main estimates (Fig. A.6). For years of schooling, we report an $R^2$ of 0.64 and a population-weighted $R^2$ of 0.69. This stability suggests that social media data can provide reliable predictions even when outcome data extend beyond the data collection period, likely reflecting the slow-moving nature of educational attainment.

### Feature importance

**Fig. 6:**
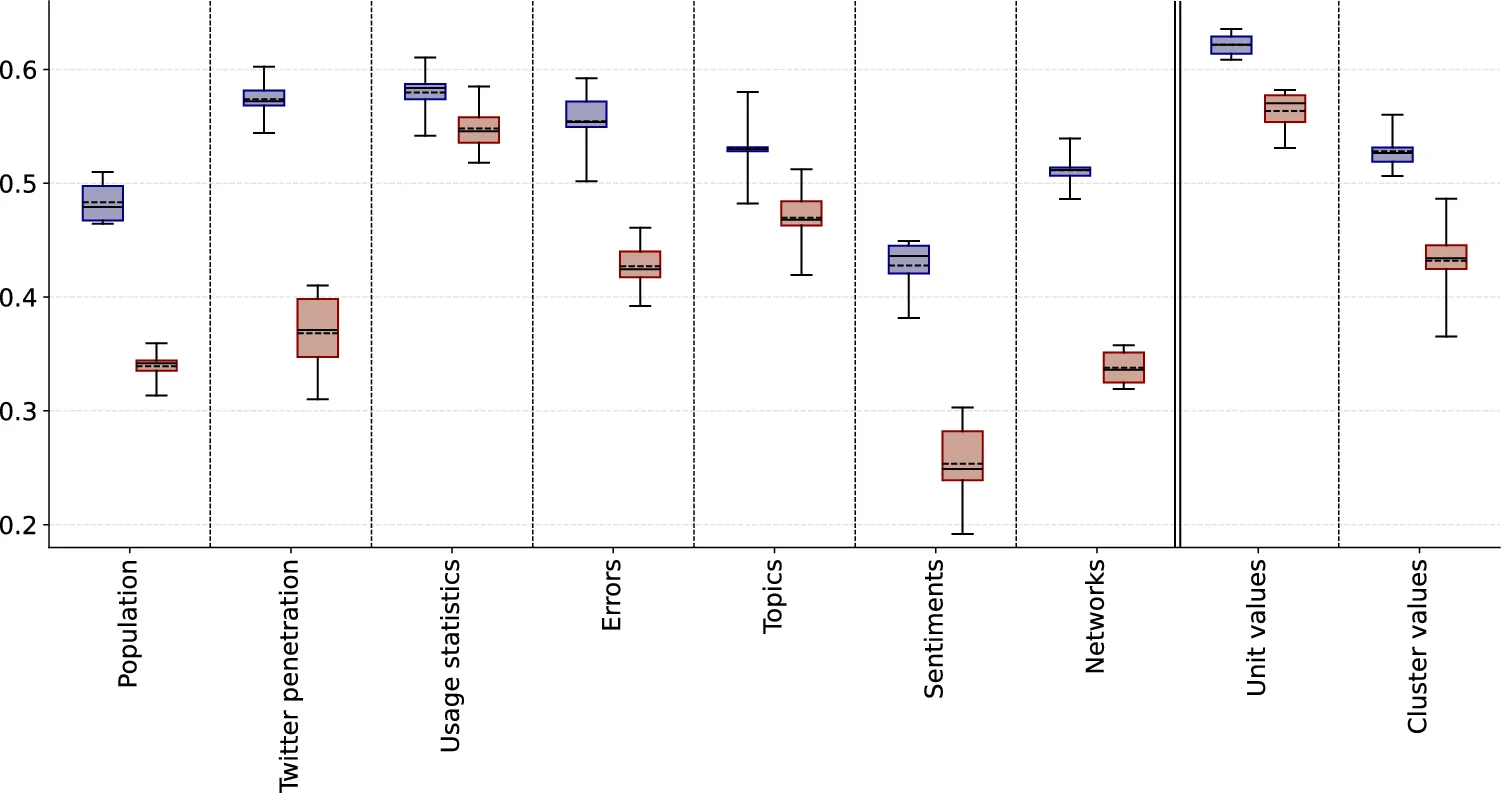
Performance of feature subgroups. Performance of feature subgroups for Mexico (blue) and the United States (red): Population (2x4 features, i.e., 4 at the unit level and 4 at the cluster level), Twitter penetration (2x4 features), usage statistics (2x11 features), spelling mistakes (MX: 2x23 features, US: 2x16 features), topics (2x19 features), sentiment (2x4 features), and networks (2x4 features), as well as all features at the unit level (i.e., municipality or county) and all features at the cluster level (i.e., including spatial neighbors). All models are evaluated through five-fold cross-validation. Boxplots show the median (solid line), mean (dotted line), the 20th & 80th percentile (box limits), as well as the minimum & maximum (whiskers) for the $R^2$ across validation folds for each outcome and country. The outcome is years of schooling in all models.

As our model is based on a limited number of interpretable inputs (see Sections C and D in the Appendix), we can explore how important various types of features are to the success of our approach. Figure 6 shows how different groups of features perform on their own. A model using only population data serves as a benchmark and achieves an $R^2$ of 0.48 for Mexico and 0.34 for the United States. Simple Twitter penetration data, that is, user and tweet densities or counts, already outperforms the population model, with $R^2$ values of 0.57 for Mexico and 0.36 for the United States. Particularly in Mexico, knowing where people tweet is thus more informative about human capital concentration than knowing where people live.

The performance of usage statistics, i.e., features such as the average tweet length or the number of followers, is high in both countries, accounting for 55 to 58 percent of the variance in educational outcomes. The same is true for topic variables, which reach an $R^2$ around 0.5 in both countries. Error and network statistics, on the other hand, seem to be much more strongly related to education in Mexico ($R^2$ of 0.55 for errors and 0.51 for networks) than in the United States ($R^2$ of 0.42 for errors and 0.34 for networks). Finally, sentiment features are the only group of variables that fails to surpass the benchmark model. We can also evaluate how our model benefited from including cluster-level features (see Figure 6). When limiting ourselves to unit-level features, we report $R^2$ values of 0.63 (MX) and 0.56 (US), as opposed to 0.70 (MX) and 0.65 (US) for the full model. This provides a lower bound for the true benefit of exploiting spatial information as cluster-level features are also used to impute missing values and extreme outliers. Thus, exploiting information from spatial neighbors is critical to the predictive power of our models. Overall, the performance of no single group of features comes close to that of the overall model, suggesting that the different inputs are complementary.

To better understand these complementarities, we use SHAP (SHapley Additive exPlanations) to estimate marginal contributions^42^. This approach is based on cooperative game theory^43^ and allows to compute importance scores for different features and feature groups (see Appendix A.2). When looking at the contributions of individual Twitter features, the user density emerges as the single important predictor in the majority of models (i.e., models for different countries and educational outcomes). It is the most important feature in five models (US: 4, MX: 1) and the second most important in the remaining (MX: 3). The importance of other features varies more strongly between countries, but topics such as sports, simple usage statistics including the tweet length or the account age, as well as network closeness centrality (only US), tend to be highly predictive too. When the SHAP values are aggregated into feature groups similar to Fig. 6, we observe that the SHAP-based feature importance ranking closely resembles the ranking based on group-specific model performance (see Appendix Section A.2 for details).

### Performance heterogeneity

**Fig. 7:**
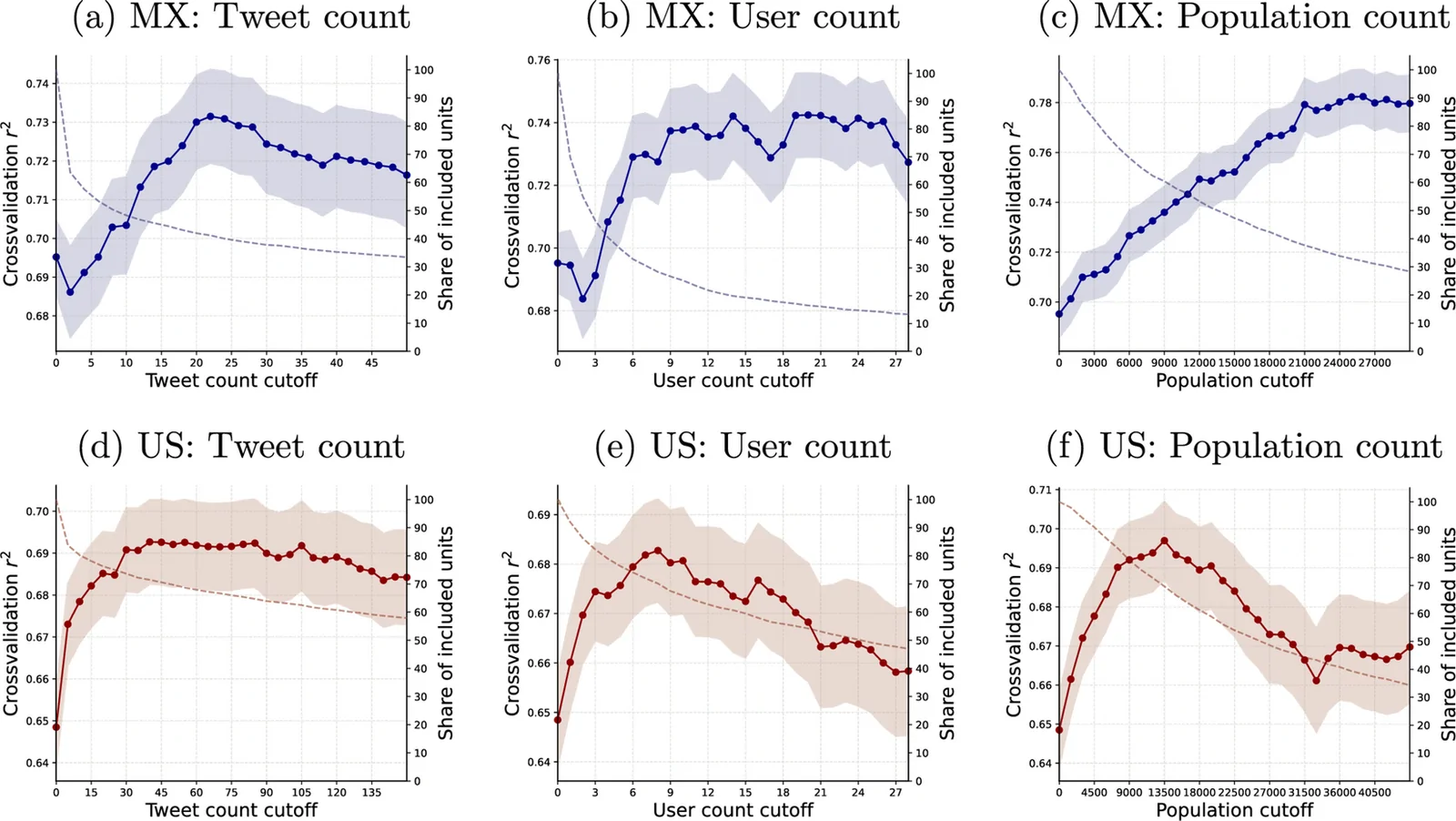
Performance heterogeneity. Performance heterogeneity by user, tweet, and population count for Mexican municipalities (blue) and US counties (red). The *solid line* shows the $R^2$ (including standard errors) for units (municipalities or counties) above different tweet, user, or population count cutoffs. Standard errors (shaded area) are computed using $\sqrt{\frac{4r^2(1-r^2)^2(n - k - 1)^2}{(n^2-1)(n+3)}}$, where *n* is the sample size and *k* is the number of features^44^. The *dashed line* shows the proportion of units included at each cutoff.

We now explore how our model is affected by the limited number of tweets in sparsely populated areas (Fig. 7). In line with expectations, performance is substantially higher when limiting the evaluation to municipalities or counties with more tweets or users. This relationship is even more pronounced when looking at different population thresholds. Particularly in Mexico, model performance increases drastically if we exclude smaller municipalities, where both input and output data is likely to be more noisy. This is consistent with finding that, in both countries, the population-weighted $R^2$ is substantially higher than the unweighted $R^2$ for all outcomes.

**Fig. 8:**
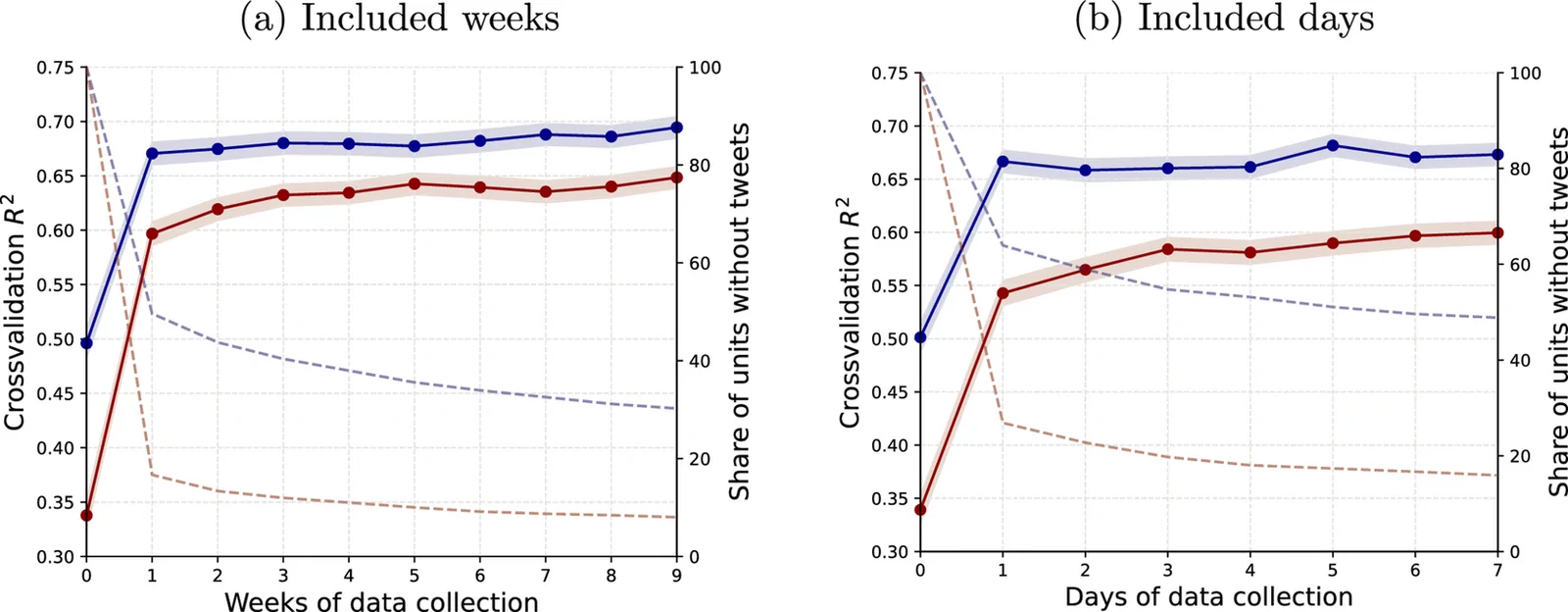
Performance by data collection period. Model performance for shorter data collection periods for Mexican municipalities (blue) and US counties (red). The value for 0 weeks/days corresponds to an $R^2$ of our baseline model using population data only. Standard errors are computed using the same formula as reported in Fig. 7.

It is also informative to look at performance by the amount of data we use to make the predictions. For our main analyses, we streamed Twitter data for two months, and used millions of tweets to construct municipality or county-level indicators. To see if similar results can be achieved with a shorter data collection period, we re-run the entire feature engineering and model training procedure on different subsets of our data. As Fig. 8 shows, drastically shortening the data collection period only marginally reduces performance. This is especially true for Mexico, where one day of tweets already yields an $R^2$ of more than 0.65. In the US, on the other hand, it takes about a week of Twitter data to account for 60 percent of the variation in county-level education outcomes. As the curves for both countries flatten out almost completely after a few weeks, extending the data collection period beyond two months is likely to yield only negligible additional performance gains.

**Fig. 9:**
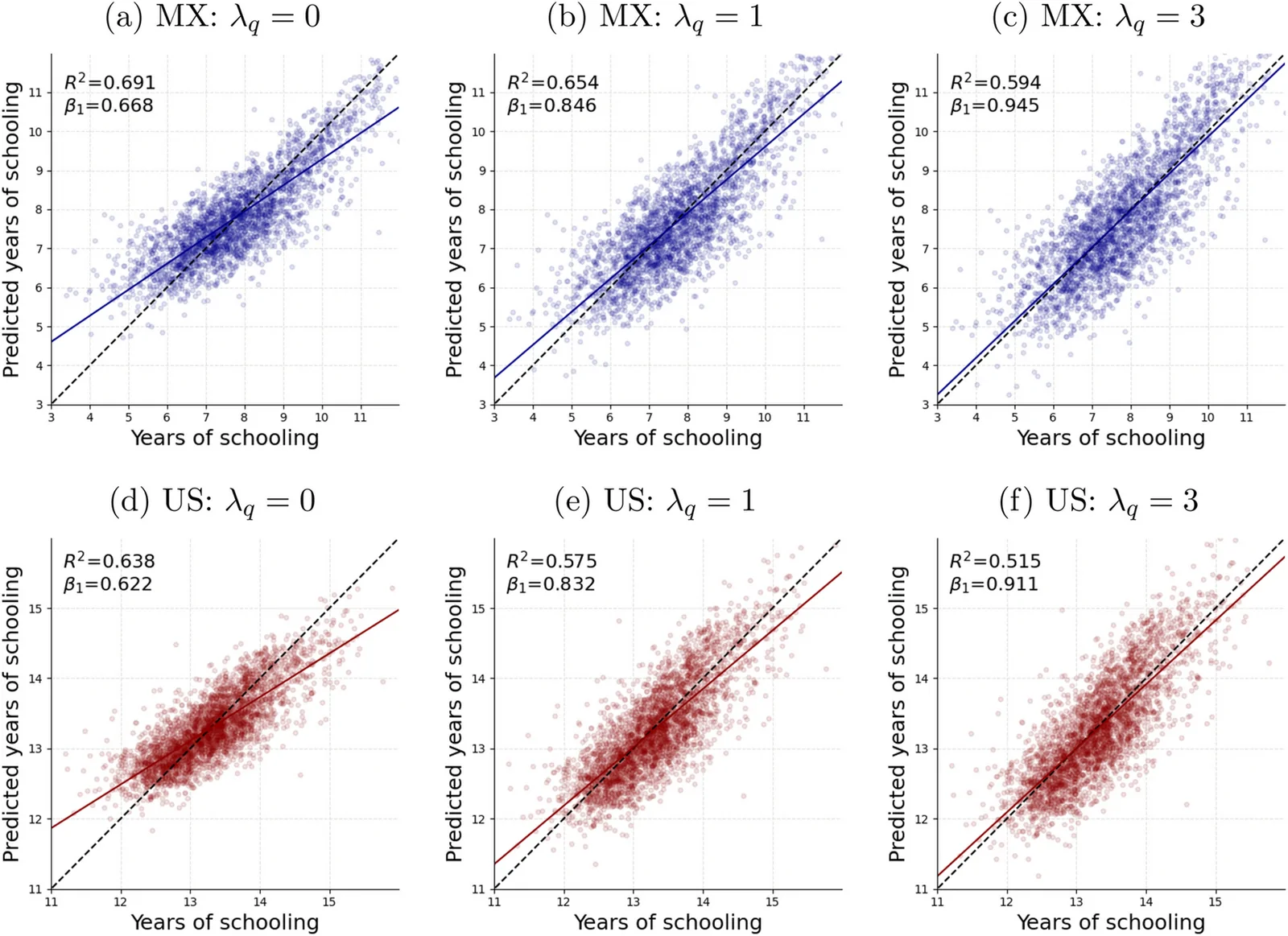
Effects of Berkson-type error correction. True vs. predicted values with correction of the Berkson-type error for Mexican municipalities (blue) and US counties (red). For the correction, we apply an adjusted loss function in the final ridge regression model that performs the stacking. Following Ratledge et al.^4^, we add an additional penalty term to the standard loss function of the ridge regression, which consists of the mean squared error (*MSE*) plus an $L_2$ penalty. The adjusted loss function is thus $MSE + \lambda _l L_2 + \lambda _q Q_{bias}$, where $\lambda _q$ is the strength of the additional penalty and a hyperparameter that can be tuned. $Q_{bias}$ is the maximum of the squared quintile-specific biases, equal to $\max _j(\mathbb {E}[\hat{y_i} - y_i|y_i \in Q_j]^2)$, where $Q_j \in \{Q_1, ...\, , Q_5\}$, and $\hat{y_i}$ is the predicted *y* for observation *i*. The figure shows the effect of three $\lambda _q$ parameters on the prediction bias. Solid lines indicate the best linear fit of each model, while dashed black lines represent the expected fit without bias ($\beta _1 = 1$).

**Table 2:** Downstream regression results.

	Mexico				United States			
	Years of Schooling	Post- Basic	Secondary	Primary	Years of Schooling	Bachelor	College	High School
$\beta _t\!:\, edu \sim econ$	0.740 (0.014)	0.661 (0.015)	0.703 (0.014)	0.728 (0.014)	0.692 (0.013)	0.707 (0.013)	0.655 (0.013)	0.487 (0.016)
$\beta _p\!:\, \widehat{edu} \sim econ$	0.549 (0.013)	0.499 (0.014)	0.526 (0.012)	0.516 (0.012)	0.496 (0.011)	0.526 (0.012)	0.470 (0.011)	0.320 (0.011)
$\beta _c\!:\, \widehat{edu_c} \sim econ$	0.748 (0.017)	0.651 (0.018)	0.744 (0.018)	0.687 (0.016)	0.699 (0.016)	0.727 (0.017)	0.661 (0.018)	0.362 (0.013)
$\beta _t - \beta _p$	−0.191 (0.012)	−0.161 (0.011)	−0.177 (0.012)	−0.212 (0.014)	−0.196 (0.011)	−0.181 (0.012)	−0.185 (0.011)	−0.167 (0.012)
$\beta _t - \beta _c$	0.008 (0.014)	−0.010 (0.013)	0.041 (0.015)	−0.042 (0.016)	0.007 (0.014)	0.021 (0.016)	0.005 (0.017)	−0.125 (0.014)
$\beta _t\!:\, econ \sim edu$	0.740 (0.014)	0.661 (0.015)	0.703 (0.014)	0.728 (0.014)	0.692 (0.013)	0.707 (0.013)	0.656 (0.013)	0.488 (0.016)
$\beta _p\!:\, econ \sim \widehat{edu}$	0.794 (0.018)	0.717 (0.019)	0.826 (0.019)	0.863 (0.019)	0.765 (0.017)	0.738 (0.017)	0.767 (0.018)	0.640 (0.023)
$\beta _c\!:\, econ \sim \widehat{edu_c}$	0.577 (0.013)	0.539 (0.015)	0.564 (0.013)	0.646 (0.015)	0.535 (0.012)	0.520 (0.012)	0.443 (0.012)	0.515 (0.019)
$\beta _t - \beta _p$	0.054 (0.012)	0.056 (0.012)	0.123 (0.013)	0.135 (0.014)	0.072 (0.015)	0.031 (0.014)	0.111 (0.014)	0.152 (0.025)
$\beta _t - \beta _c$	−0.162 (0.010)	−0.122 (0.011)	−0.139 (0.011)	−0.083 (0.012)	−0.157 (0.013)	−0.187 (0.012)	−0.212 (0.012)	0.028 (0.024)
N	2,457	2,457	2,457	2,457	3,140	3,140	3,140	3,140

The predictions for different educational outcomes, referred to as *edu*, are denoted as $\widehat{edu}$, and *econ* is wealth for Mexico and income for the United States. For $\widehat{edu}_c$, we apply a Berkson error correction with $\lambda _q$= 3 for years of schooling and $\lambda _q$= 15 for all other outcomes (i.e., all percentages). Results are reported in standard deviations ($\widehat{edu}$ and $\widehat{edu}_c$ are standardized using the distribution of *edu*).$\beta _t-\beta _p$ is the original bias and $\beta _t-\beta _c$ is the bias using the predictions based on the adapted loss function. Education is the dependent variable in the top panel and the independent variable in the bottom panel. The bias becomes insignificant for 5 out of 8 outcomes. The correction appears to be particularly effective for outcomes that have a higher initial $R^2$. In the last model (high school), which is also the one with the lowest initial $R^2$, the penalized loss function achieves only a limited slope correction with $\lambda _q = 15$ (not shown) and the regression is thus unable to recover the true effect. Standard errors in parentheses.

### Downstream performance

In addition to being directly useful to better understand local patterns in development outcomes and target interventions accordingly, predicted measures may also serve to study relationships with other variables. Using wealth data for Mexico and income data for the United States (see Appendix Table D.1), we thus explore how our Twitter-based indicator performs in downstream regression tasks. The fact that machine-learning-derived indicators are noisy measures gives rise to several potential biases that can compromise such applications. If *edu* is the true distribution of the indicator we predicted as $\widehat{edu}$ (e.g., years of schooling), and *econ* is another variable whose relationship to *edu* we wish to study (e.g., wealth), three types of measurement error may occur (see simulations in Appendix Figure B.1):

1. Attenuation bias: A random measurement error in $\widehat{edu}$ will dilute the correlation between *edu* and *econ*. This results in an attenuation bias when regressing *econ* on $\widehat{edu}$, but not in the opposite specification, and decreases precision in both cases (see, e.g., Fuller^45^).
2. Berkson-type error: A bias that has only recently gained attention^4^ arises when measurement errors are correlated with *edu*. The typical behavior of machine learning models is to overpredict for low values and underpredict for high values, a pattern that is very apparent in our application, where the correlation between the prediction error (i.e., $\widehat{edu}$ - *edu*) and *edu* amounts to about −0.6. This does not affect the correlation between *edu* and *econ*, but it distorts coefficients in downstream regressions. Specifically, it leads to a downward bias when $\widehat{edu}$ is used as the outcome variable, and to an upward bias when it acts as the explanatory variable.
3. Correlated learning: If the features used to predict $\widehat{edu}$ contain wealth or income-related information, our model might exploit the correlation between *econ* and *edu* to make better predictions. Given that some of our features, such as Twitter penetration, usage and network features, are likely related to both education and broader socioeconomic conditions, this is to be expected in our setting. Indeed, our feature matrix is almost as predictive of economic outcomes ($R^2$ = 0.64 for wealth in Mexico and $R^2$ = 0.62 for income in the US) as of education. This is substantially higher than a model using education only (years of schooling) for the prediction (MX: 0.57, US: 0.50), suggesting that our feature matrix indeed contains wealth- and income-related information that is independent of education levels. This creates an artificially strong correlation between $\widehat{edu}$ and *econ*. When using $\widehat{edu}$ as the dependent variable, this only leads to overoptimistic standard errors. If $\widehat{edu}$ is the independent variable (and *edu* and *econ* are positively correlated), it additionally induces an upward bias for the point estimate.

With these considerations in mind, we now compare the downstream correlations (Appendix Figure B.2) and regression results (Table 2) of $\widehat{edu}$ and *econ* with the true correlations captured by *edu*. As Figure B.2 in the Appendix shows, the predicted education indicator consistently understates true correlations, suggesting that the attenuation bias dominates over a potential bias due to correlated learning. Table 2 further shows that the slope of the regression coefficients is considerably underestimated for all outcomes when using $\widehat{edu}$ as the dependent variable of the regression and slightly overestimated in the reverse specification, a pattern that is consistent with a Berkson-type error. Hence, it appears that the correlation estimates are mainly affected by attenuation, while biases in regression coefficients are largely driven by a Berkson-type error.

While the attenuation bias and correlated learning cannot be avoided in most applications, it is possible to refine our model in a way that minimizes the Berkson error. Following Ratledge et al.^4^, we add a further penalty term for a quintile-specific bias to the loss function of our final stacking model. If the weight given to this penalty is sufficiently high, the tendency to understate high values and overstate low values effectively disappears (see Fig. 9), albeit at the cost of reduced overall performance, with a decrease in the $R^2$ by about 10 percentage points. Using this new set of predictions (see Table 2), the bias in the top panel ($\widehat{edu}\sim econ$) becomes negligible for most outcomes. In the bottom panel ($econ\sim \widehat{edu}$) the direction of the bias is reversed as the attenuation bias begins to dominate. This suggests that when appropriately modeled, predicted indicators can yield accurate estimates in downstream regression tasks, provided they serve as the outcome rather than the treatment variable. Fortunately, the former scenario is more common, as it enables the evaluation of interventions or policy changes.

## Conclusion

Our results show that human capital can be accurately inferred from Twitter data using machine learning. We are able to account for up to 70 percent of the variation in years of schooling in Mexico and up to 65 percent in the United States. This is substantially higher than the performance reported in previous attempts to predict human capital, and comparable to the effectiveness of satellite data in predicting wealth. Performance remains strong when assessed through the more stringent leave-one-state-out cross-validation rather than five-fold cross-validation, confirming that our approach generalizes to entirely new geographic areas. As only a few days of Twitter data are needed to achieve a good performance and the natural language processing tools we use for feature preparation support many different languages, our approach has substantial potential for broader application. In addition, despite the lower Twitter penetration, our model tends to perform better for Mexico than for the United States, suggesting that the method is also relevant to less affluent regions with lower levels of social media usage.

In addition to being directly useful for understanding spatial patterns and targeting interventions, predicted indicators also have the potential to advance scientific research by providing inputs for downstream inference tasks. This paper highlights that such applications are not without caveats. Our data and simulations show that estimates in downstream regression tasks tend to be subject to several biases. We further demonstrate that these biases can be corrected using an adapted loss function (see Ratledge et al.^4^) if the predicted indicator serves as the dependent variable. If carefully tuned, machine-learning-derived indicators can thus become a valuable data source to study effects on outcomes for which ground truth data are unavailable. However, more research is needed to better understand the empirical relevance of each of the biases, and experiment with the most effective ways to address them.

These contributions notwithstanding, three important limitations should be acknowledged. *First*, we report performance of our approach for a specific platform at a specific point in time, namely Twitter in 2021. Twitter (now X) data has since changed substantially, with tighter restrictions and higher costs for large-scale data collection. In parallel, the platform has undergone shifts in governance, user composition, and posting behavior^39–41^. While the data we use remain highly predictive of today’s education levels, real-time prediction or tracking changes over time would require recalibrating the model to the current Twitter environment or to a different platform. *Second*, our evaluation is limited to two countries. Although the United States and Mexico differ substantially in economic development, language, and Twitter penetration, we cannot make precise predictions regarding transferability to other countries, especially those with very low platform adoption. The strong performance in Mexico, which has relatively low Twitter penetration compared to other countries in the Americas, suggests our approach may be applicable beyond high-penetration contexts, but further research is needed to confirm this. *Third*, within countries, Twitter data is less informative at the lower end of the education distribution and in less populated areas with lower Twitter penetration. This likely reflects that Twitter use is concentrated among the highly educated, making the platform less suited for distinguishing between low and medium education levels. Including data from other platforms with less selective usage patterns may be a promising avenue for future research.

Looking forward, recent advances in large language models present both interesting possibilities and challenges for our approach. On the one hand, LLMs could enable more sophisticated evaluation of tweet content, including advanced error detection, zero-shot topic classification, and text embeddings as features, potentially increasing predictive performance and facilitating multi-language integration. On the other hand, as social media users increasingly adopt AI writing assistants, education signals from indicators such as spelling errors may become diluted. Nevertheless, most feature groups, such as penetration, usage statistics, network structures, and topics, should remain informative, and differential AI adoption across demographic groups^46,47^ may create new predictive signals.

Overall, our results demonstrate that social media data can provide accurate spatially granular estimates of educational attainment. As social media use continues to expand globally, this approach offers an increasingly promising complement to traditional survey methods for measuring human capital development.

## Supplementary Information

Supplementary Information.

## Data Availability

The datasets generated and analyzed in this study are available in the Harvard Dataverse repository at https://doi.org/10.7910/DVN/CWS2BJ.

## Code availability

The code used to generate the results reported in this study is available in the Harvard Dataverse repository at https://doi.org/10.7910/DVN/CWS2BJ.
