# Supplementary material for: Measuring human capital with social media data and machine learning
Source: Sci Rep. 2026 Jun 4;16:26096. doi: 10.1038/s41598-026-53076-4 (PMC13490463; doi:10.1038/s41598-026-53076-4)
Supplement: Supplementary file 1 [file 41598_2026_53076_MOESM1_ESM.pdf]

Supplementary Materials for

Measuring Human Capital with Social Media Data and Machine Learning

Martina Jakob*

University of Zurich
Department of Economics
Zurich, Switzerland
martina.jakob@econ.uzh.ch

and

University of Bern
Department of Social Sciences
Bern, Switzerland
martina.jakob@unibe.ch

Sebastian Heinrich

ETH Zurich
Department of Management, Technology, and Economics
Zurich, Switzerland
heinrich@kof.ethz.ch

May 20, 2026

Keywords: Machine Learning, Social Media Data, Education, Human Capital, Indicators, Natural Language Processing

Classification: Economic Sciences, Social Sciences

***Corresponding Author:** Martina Jakob, martina.jakob@econ.uzh.ch, University of Zurich, Department of Economics, Zurich, Switzerland

A Model Performance

A.1 Main Results

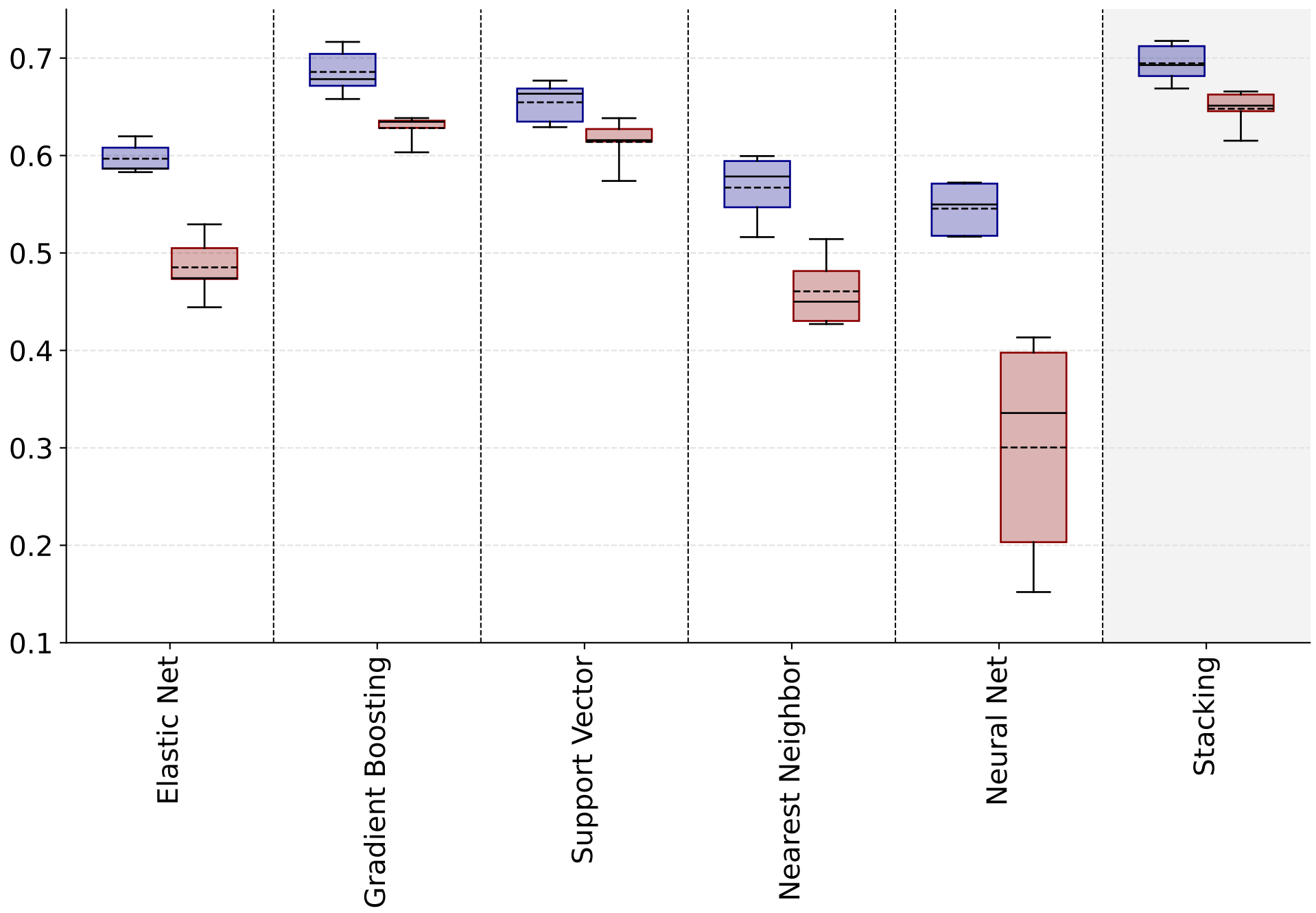

Figure A.1: Performance of individual models

Performance of individual models considered in the final stacking model for years of schooling in Mexico (blue) and the United States (red). All models are evaluated through five-fold cross-validation. Boxplots show the median (solid line), mean (dotted line), the 20th & 80th percentile (box limits), as well as the minimum & maximum (whiskers) for the R^2 across validation folds for each outcome and country.

Table A.1: Performance of individual models

	Mexico				United States			
	Years of Schooling	Post Basic Education	Secondary Education	Primary Education	Years of Schooling	Bachelor Degree	Some College	Only High School
Elastic Net	0.597<br>(2.8%)	0.621<br>(-5.6%)	0.548<br>(-15.3%)	0.495<br>(-17.7%)	0.485<br>(0.2%)	0.522<br>(-1.4%)	0.461<br>(4.6%)	0.350<br>(-4.0%)
Gradient Boosting	0.686<br>(56.9%)	0.689<br>(61.6%)	0.638<br>(62.1%)	0.600<br>(60.7%)	0.628<br>(56.5%)	0.674<br>(52.7%)	0.603<br>(55.6%)	0.467<br>(53.0%)
Support Vector Machine	0.655<br>(29.1%)	0.602<br>(6.2%)	0.544<br>(7.7%)	0.459<br>(-0.1%)	0.614<br>(41.0%)	0.669<br>(37.7%)	0.576<br>(37.1%)	0.457<br>(42.7%)
Nearest Neighbour Matching	0.567<br>(0.6%)	0.576<br>(-0.2%)	0.523<br>(7.5%)	0.490<br>(11.5%)	0.461<br>(3.3%)	0.504<br>(0.2%)	0.425<br>(3.3%)	0.359<br>(15.2%)
Multi-layer Perceptron	0.545<br>(13.2%)	0.654<br>(40.3%)	0.590<br>(41.4%)	0.557<br>(48.1%)	0.300<br>(6.2%)	0.627<br>(16.9%)	0.424<br>(6.7%)	-0.523<br>(3.2%)
Stacking	0.695	0.689	0.638	0.607	0.648	0.697	0.620	0.500

Mean R^2 and stacking weights (in parentheses) across folds for different models and outcomes. Note that R^2 values for the final stacking model reported as our main results are computed using the combined out-of-sample predictions of all folds rather than as the mean across folds, and may thus slightly differ.

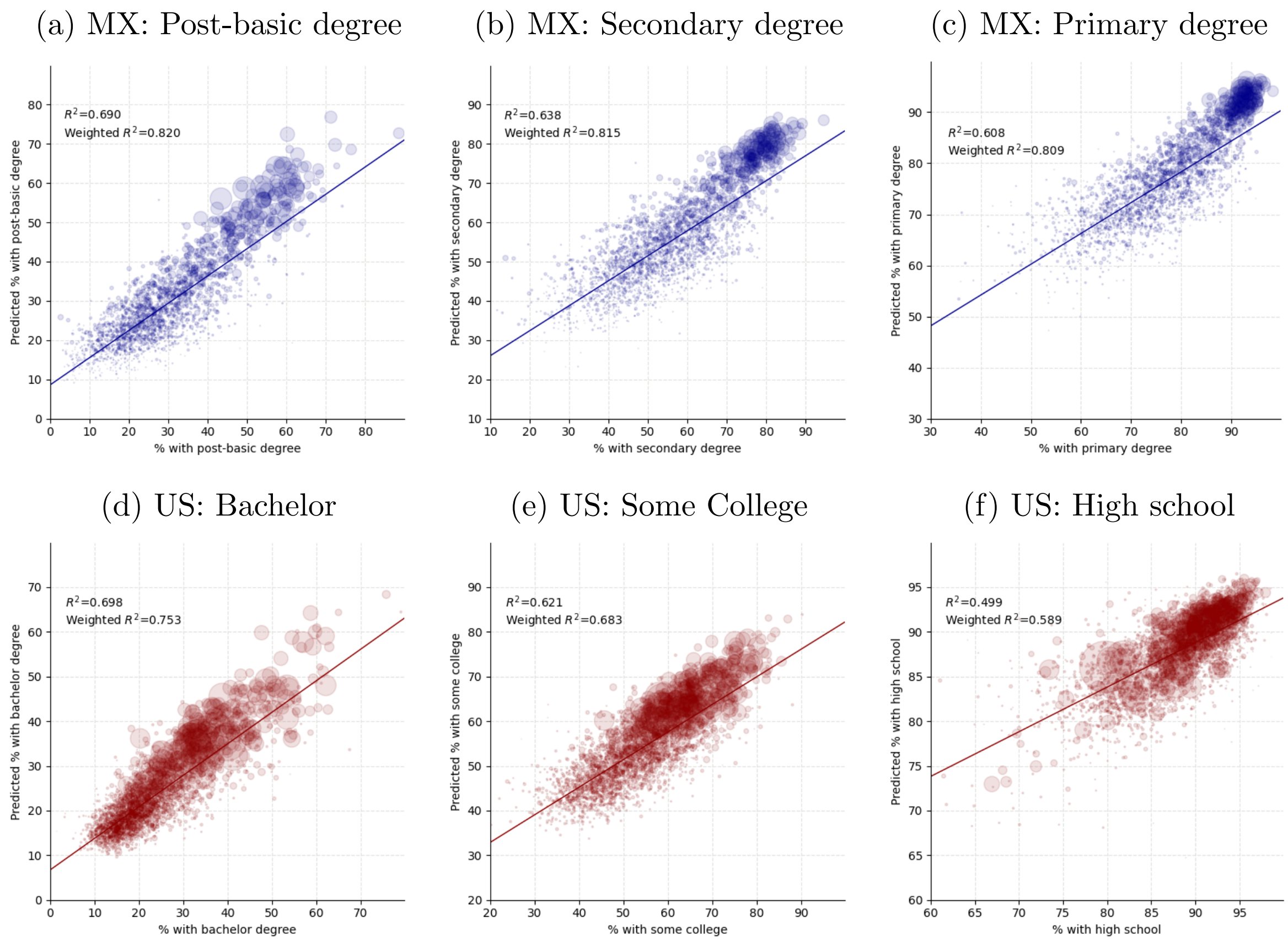

Figure A.2: True vs. predicted values for secondary outcomes (5-fold)

Predicted values for all Mexican municipalities (blue) and US counties (red) are obtained by combining out-of-sample predictions from five-fold cross-validation. Bubble size is proportional to the population in each unit. R^2 and population weighted R^2 shown. Line indicating best linear fit is not population weighted.

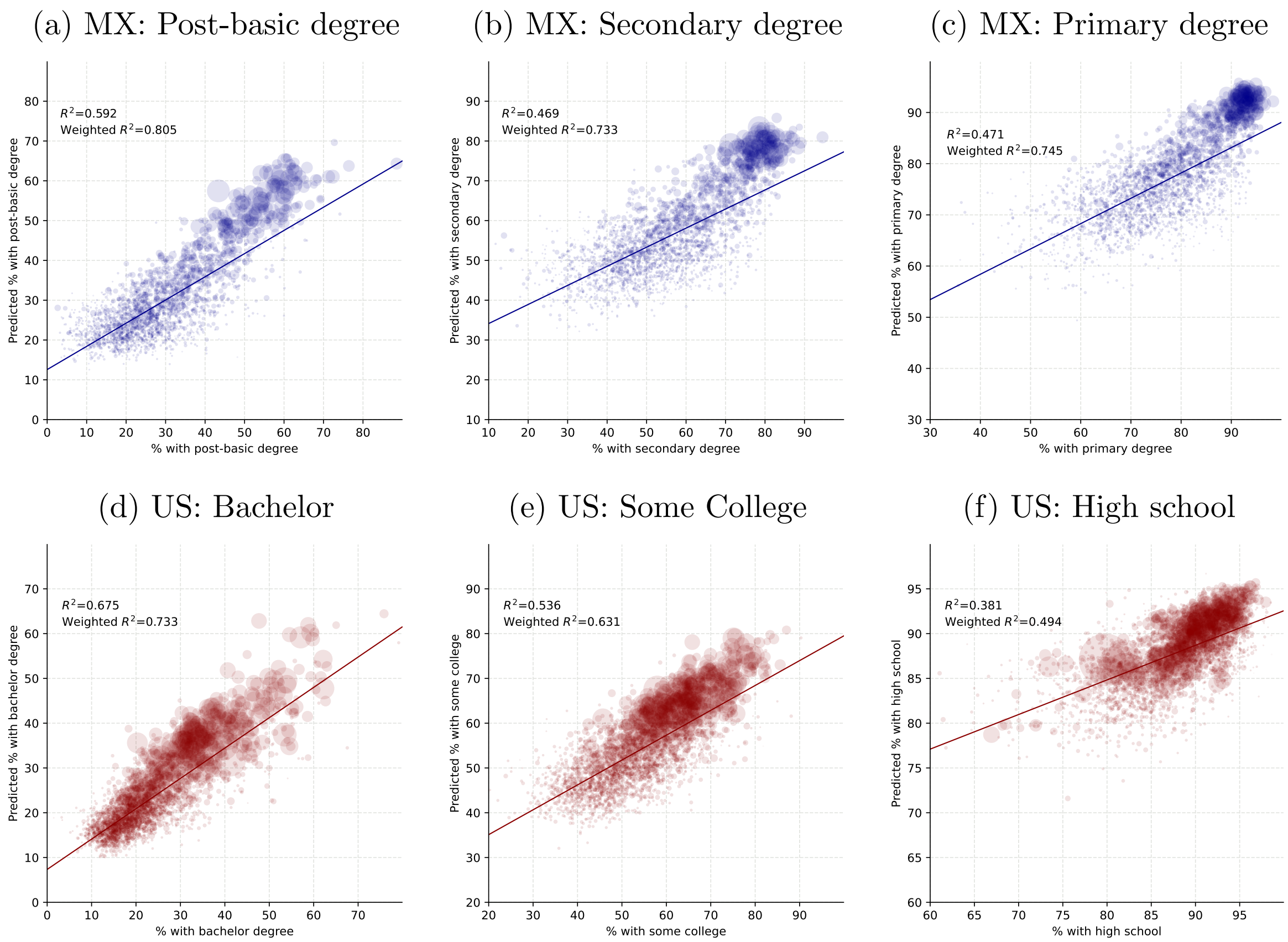

Figure A.3: True vs. predicted values for secondary outcomes (LOSO)

Predicted values for all Mexican municipalities (blue) and US counties (red) are obtained by combining out-of-sample predictions across all held-out states from leave-one-state-out (LOSO) cross-validation. Bubble size is proportional to the population in each unit. R^2 and population-weighted R^2 are shown. Line indicating best linear fit is not population-weighted.

A.2 Feature Importance

We compute SHAP values from the final stacking regressor, which combines predictions from five base models (elastic net, gradient boosting, support vector regression, nearest neighbor regression, and neural network) and is thus representative of our overall approach. SHAP values are calculated for each municipality or county based on out-of-sample predictions from cross-validation. We present results for the most important individual features (Figure A.4a) as well as per group aggregations (Figure A.4b) based on sums of absolute SHAP contributions. Note that while for signed SHAP values, the sum of feature contributions equals the difference between the individual prediction and the average prediction across all observations, this "additivity property" does not hold for

(a) Most important features

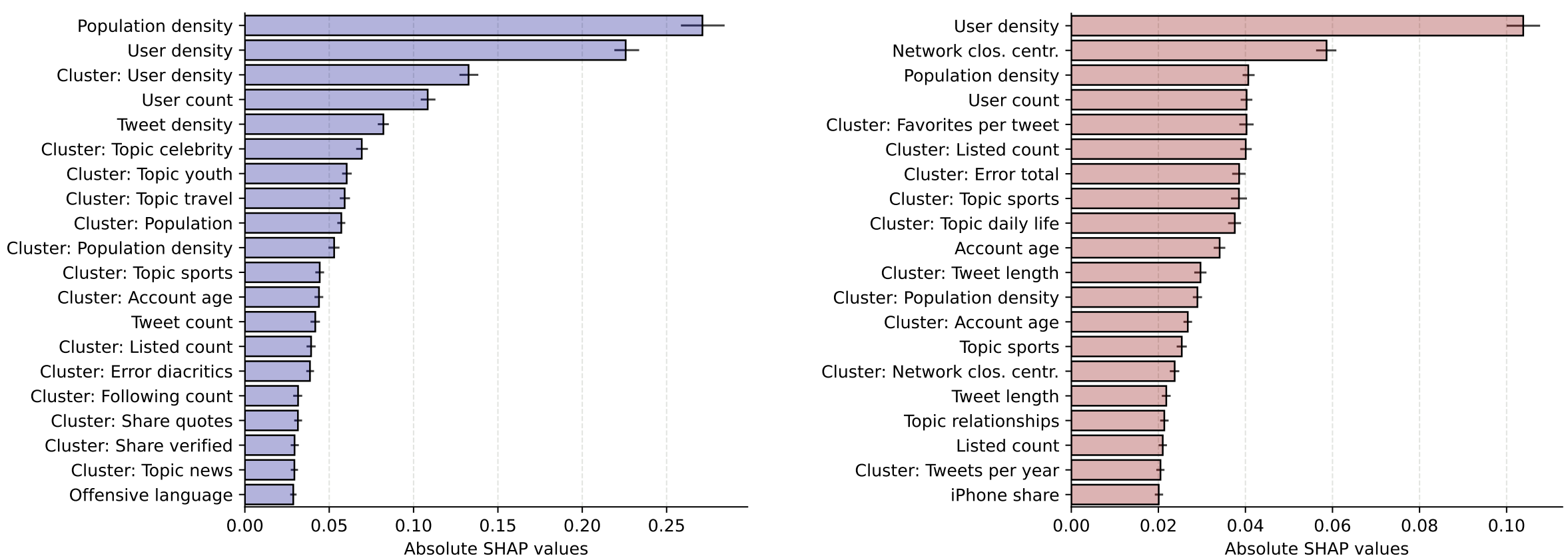

(b) Aggregation by feature subgroups

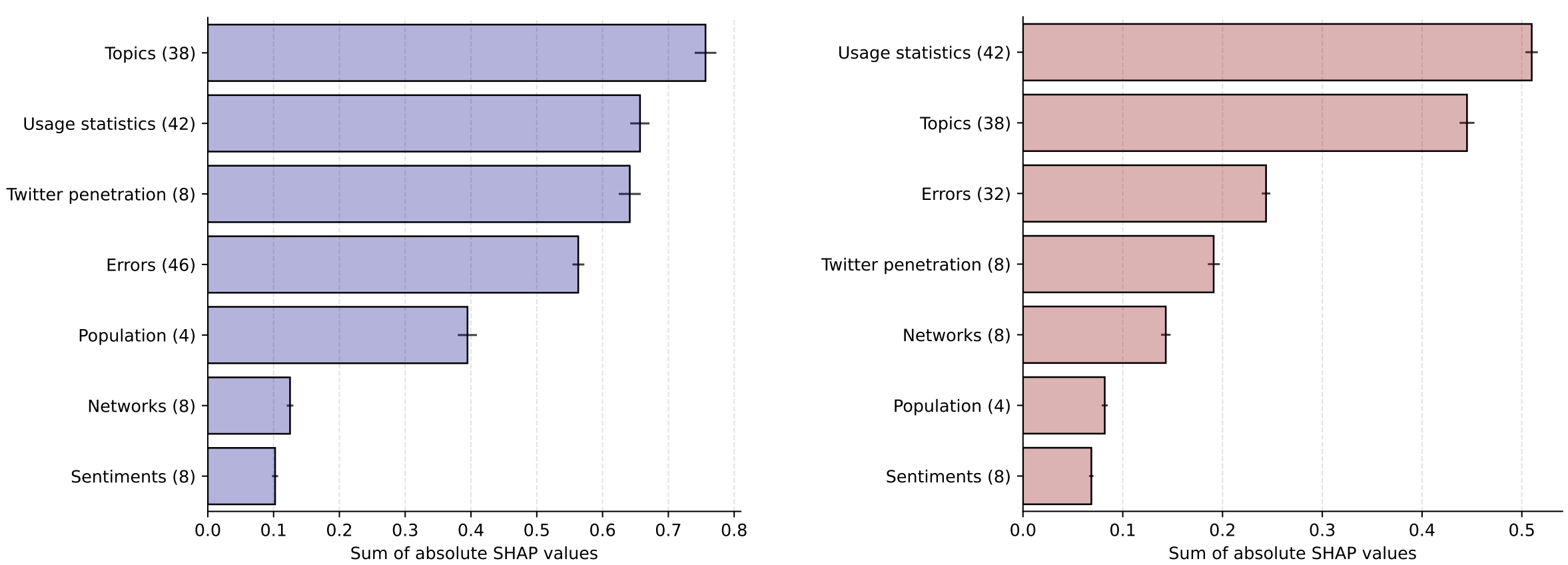

Figure A.4: SHAP value feature importance for stacking regressor

Most important features and feature subgroups in the stacking regressor for Mexico (blue) and the United States (red). The displayed feature importance is based on SHAP (SHapley Additive exPlanations) values, an approach based on cooperative game theory (Lundberg and S.-I. Lee, 2017). In Figure A.4a, the 20 most important features are shown for each country, ranked according to the absolute SHAP value. Figure A.4b presents the sum of absolute SHAP values for 7 different feature groups. Note that due to the high correlation between some features, estimates should be interpreted with caution.

absolute values (Figure A.4a) or sums of absolute values (Figure A.4b). Our presented measures should thus be interpreted as heuristic summaries of predictive contribution rather than formal decompositions.

While SHAP is specifically designed to assess contributions of correlated individual features, results could still be unstable under very high multicollinearity. We assess robustness in two steps. First, we examine pairwise feature correlations in our feature matrices, finding that only a very limited number of feature pairs are highly correlated (see Figure A.5), with mean absolute correlations of $|r| = 0.16$ for Mexico and $|r| = 0.15$ for the United States. Second, to directly assess whether feature importance rankings are stable across different model specifications and data samples, we examine the consistency of SHAP values across cross-validation folds. Each fold represents a different subsample

(a) Distribution of pairwise correlations

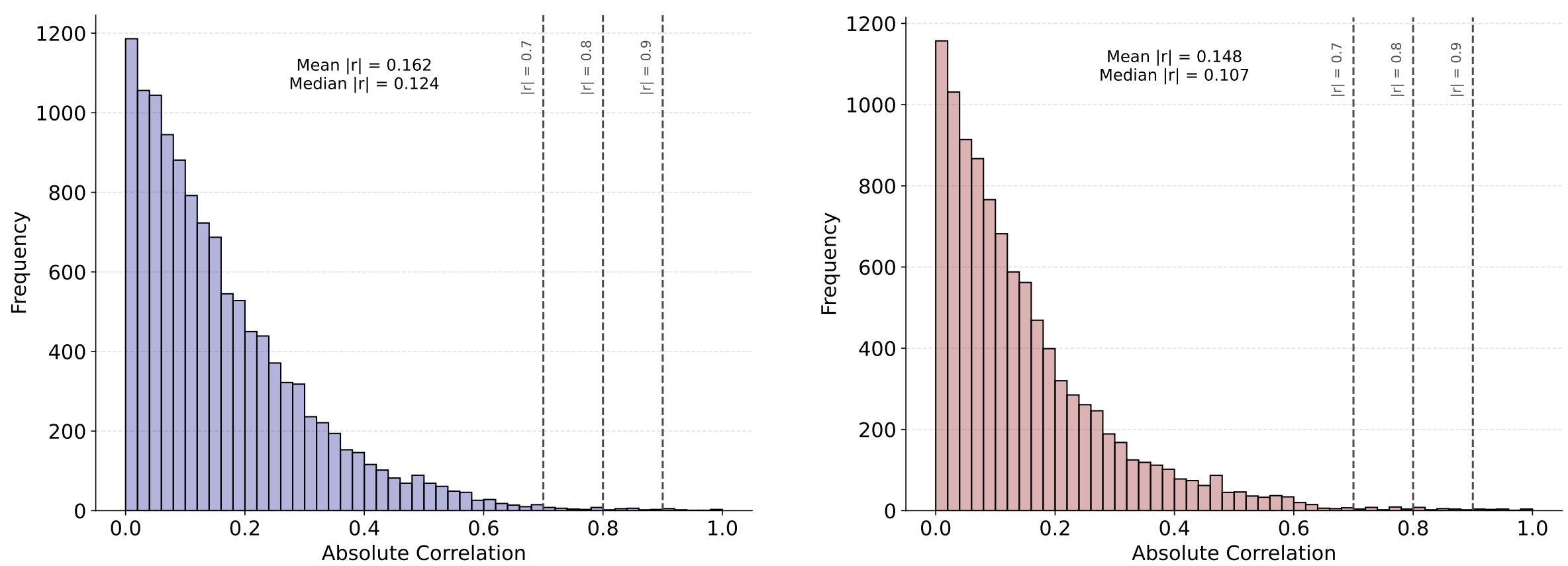

(b) High-correlation pairs by feature group

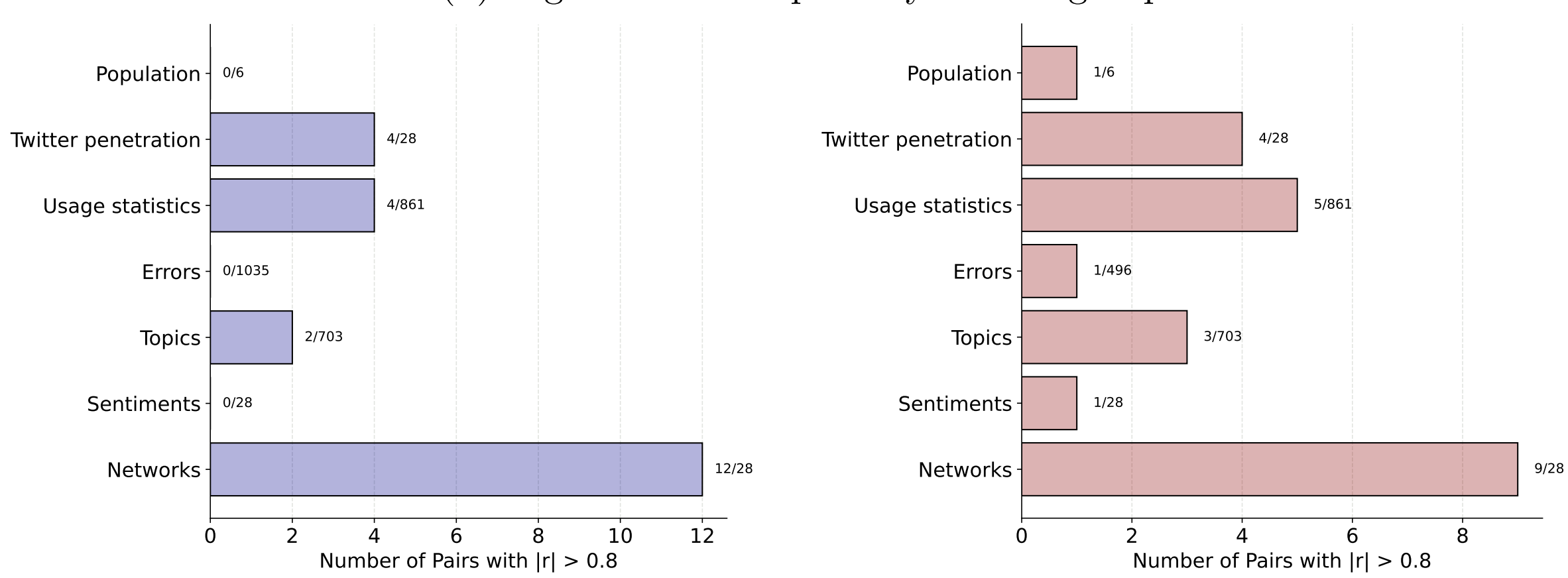

Figure A.5: Feature correlation analysis

Pairwise correlations between features for Mexico (blue) and the United States (red). Figure A.5a shows the distribution of absolute correlation coefficients across all feature pairs. Vertical dashed lines indicate thresholds at $|r| = 0.7$, 0.8, and 0.9. Figure A.5b displays the number of highly correlated feature pairs ($|r| > 0.8$) within each feature group, shown as fractions of total possible pairs. High correlations occur predominantly within network groups, while all other groups show few or no high-correlation pairs in both countries.

of municipalities/counties and a distinct model configuration with fold-specific hyperparameters and weights. Feature importance rankings prove remarkably stable, with mean Spearman rank correlations of $\rho = 0.69$ for Mexico and $\rho = 0.81$ for the United States. In both countries, the most important feature overall also consistently ranks first across all five folds. Among the top five features, 92% rank within the top five in each individual fold for Mexico and 68% for the United States, while all of them rank within the top ten in both countries.

A.3 Model Predictions for Future Outcomes

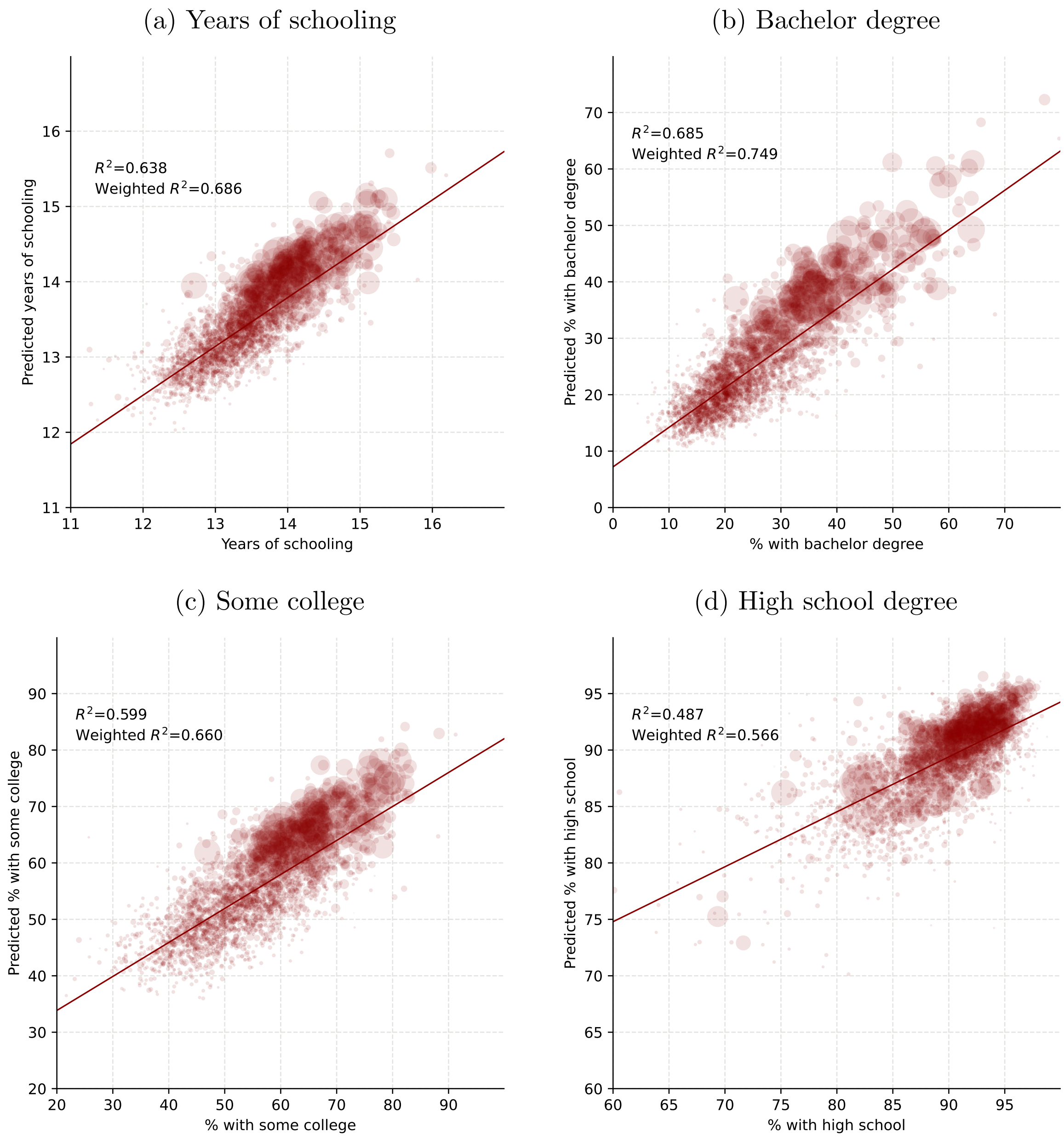

Figure A.6: Future education outcome results for the United States

Predictions vs. true values based on a model retrained and evaluated using more recent American Community Survey data (2019–2023) compared to the main analysis (2017–2021). Bubble size is proportional to population. R^2 and population weighted R^2 shown. Line indicating best linear fit is not population weighted.

A.4 Prediction Uncertainty

To assess the reliability of our predictions, we quantify prediction variability by repeatedly re-running the full cross-validation procedure with different random fold assignments. Specifically, we re-estimate the model 20 times using different random seeds for fold construction and compute, for each municipality or county, the standard deviation of the resulting out-of-sample predictions. This allows us to characterize prediction uncertainty at the unit level and examine how it correlates with observable characteristics.[1]

Uncertainty is generally higher in Mexican municipalities than in US counties (Figure A.7). This likely reflects the higher variance in years of schooling in Mexico (SD = 1.49 for Mexico vs. SD = 0.66 in the US). Within both countries, variability is substantially higher in less populous municipalities or counties, in areas with fewer Twitter users and tweets, and where the model relies more heavily on spatial imputation (Figure A.8). In Mexico, prediction variability additionally increases at lower levels of educational attainment, a pattern that is much less pronounced in the United States.

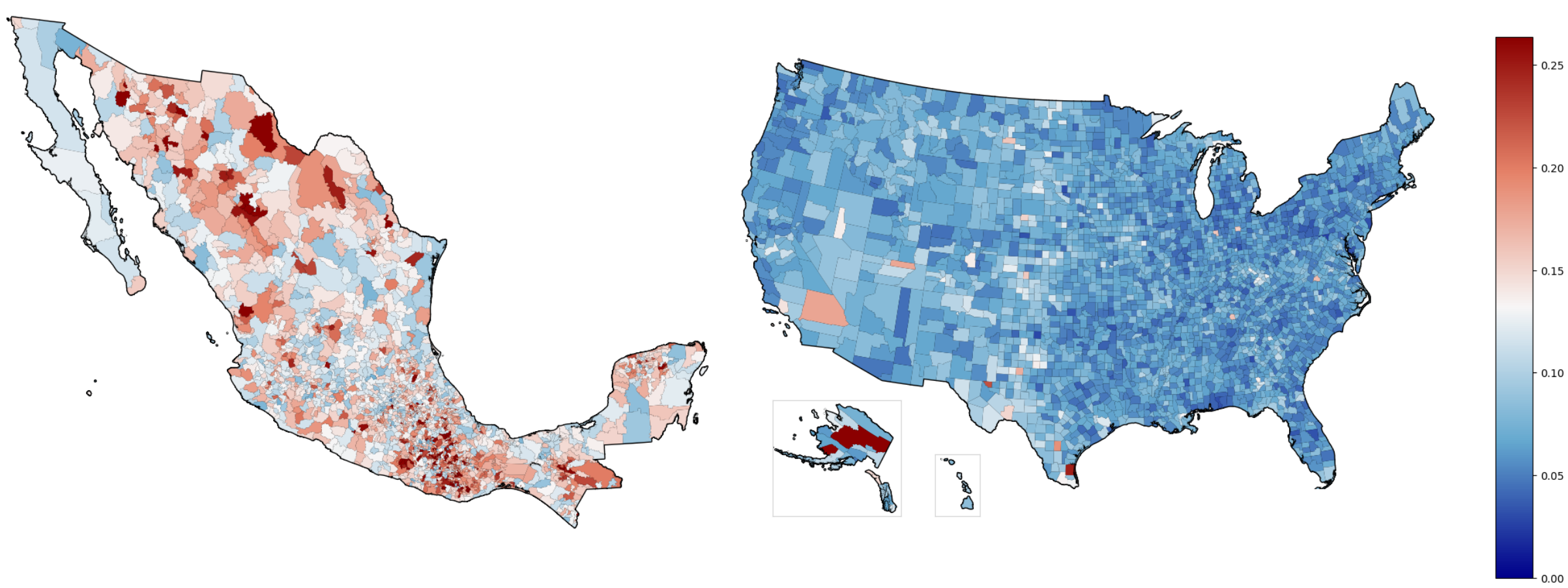

Figure A.7: Prediction uncertainty for Mexican municipalities and US counties

Prediction uncertainty for years of schooling, measured as the standard deviation of predicted values across repeated cross-validation re-runs, for each municipality in Mexico (left) and county in the United States (right).

[1]This simple approach is consistent with our overall training setup and provides an easy-to-interpret measure of prediction stability, but is computationally intensive due to repeated model re-estimation. More recent approaches to predictive uncertainty estimation include Bayesian machine learning methods, quantile-based models, and conformal prediction (e.g., Maddox et al., 2019; Akrami et al., 2022; Angelopoulos and Bates, 2021).

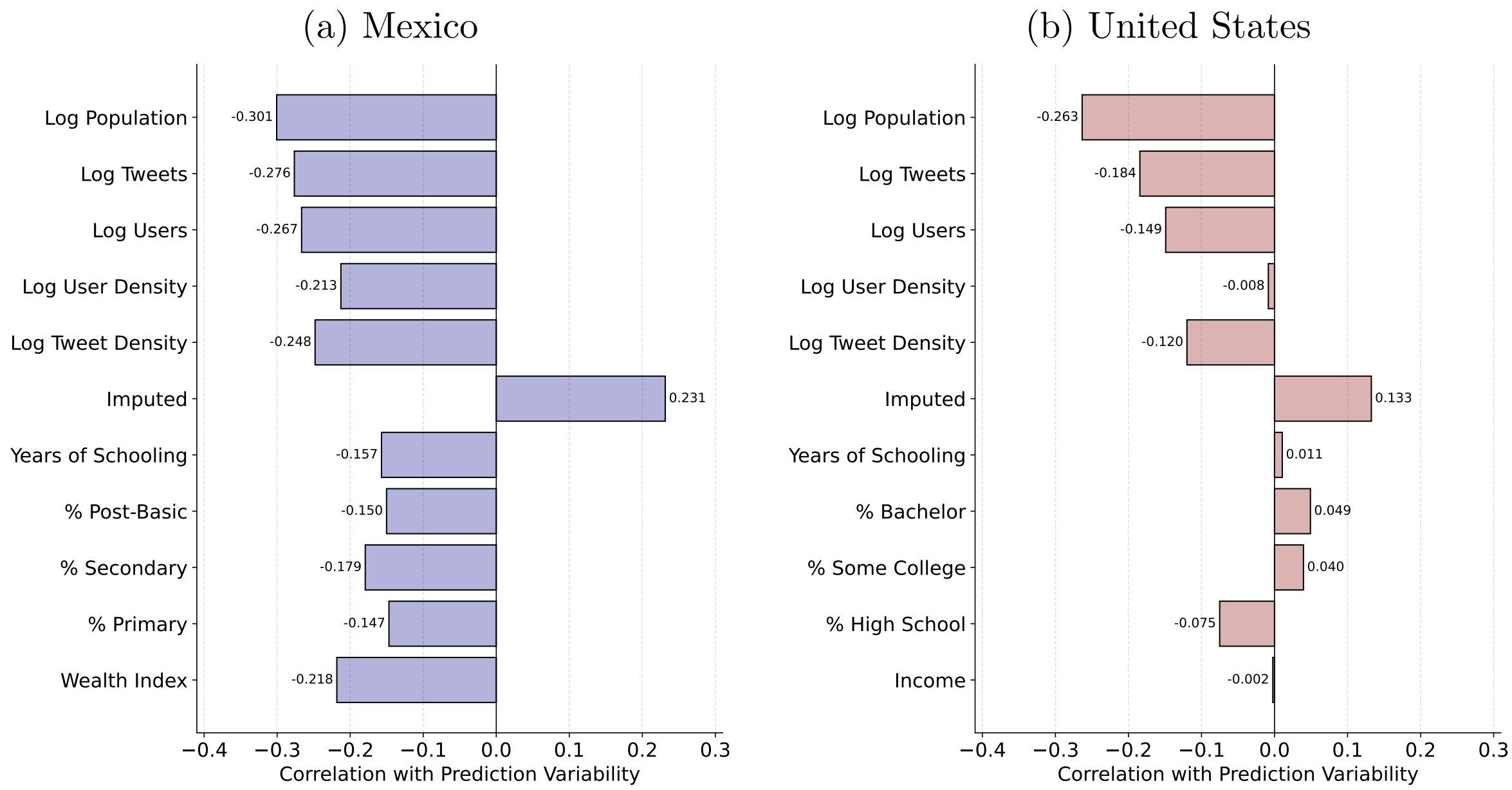

Figure A.8: Correlates of prediction variability

Unconditional correlation between prediction uncertainty for years of schooling, measured as the standard deviation of predicted values across repeated cross-validation re-runs, and selected characteristics of Mexican municipalities and US counties.

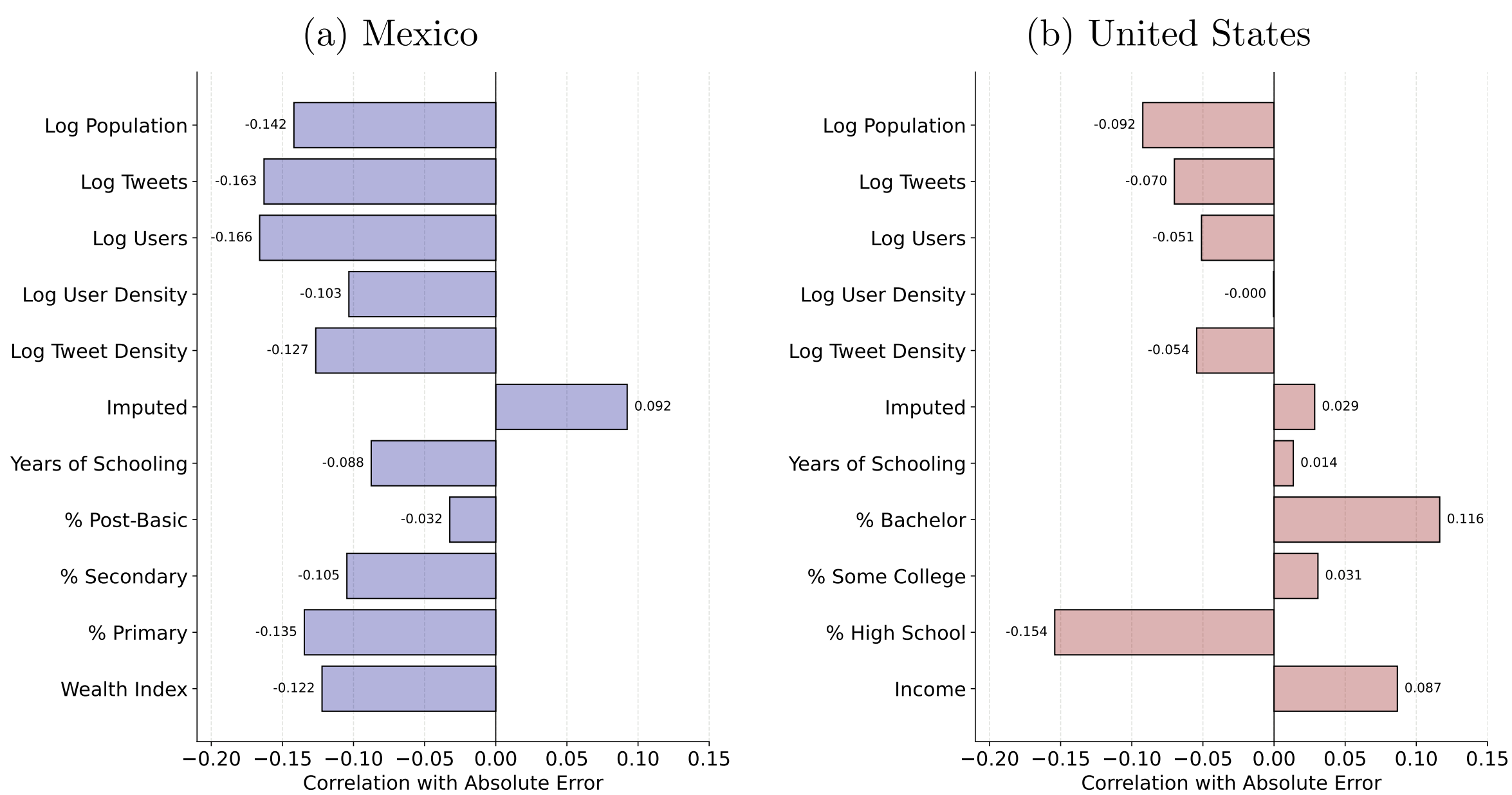

Figure A.9: Correlates of prediction error

Unconditional correlation between absolute prediction errors for years of schooling and selected characteristics of Mexican municipalities and US counties.

Relatedly, we examine correlates of absolute prediction errors. Unsurprisingly, results are similar to uncertainty, suggesting that areas with higher prediction variability also tend to have larger prediction errors (see Figure A.9). Given that spatial clustering is a key component of our data preparation pipeline, we also disaggregate performance by imputation status. As expected, we find larger mean average prediction errors (MAE) for areas that rely more heavily on imputed features. However, increases in MAE compared to non-imputed areas are only 10 percent for the US and 20 percent for Mexico, suggesting that predictions remain reasonably accurate even in these sparse-data contexts (Figure A.10).

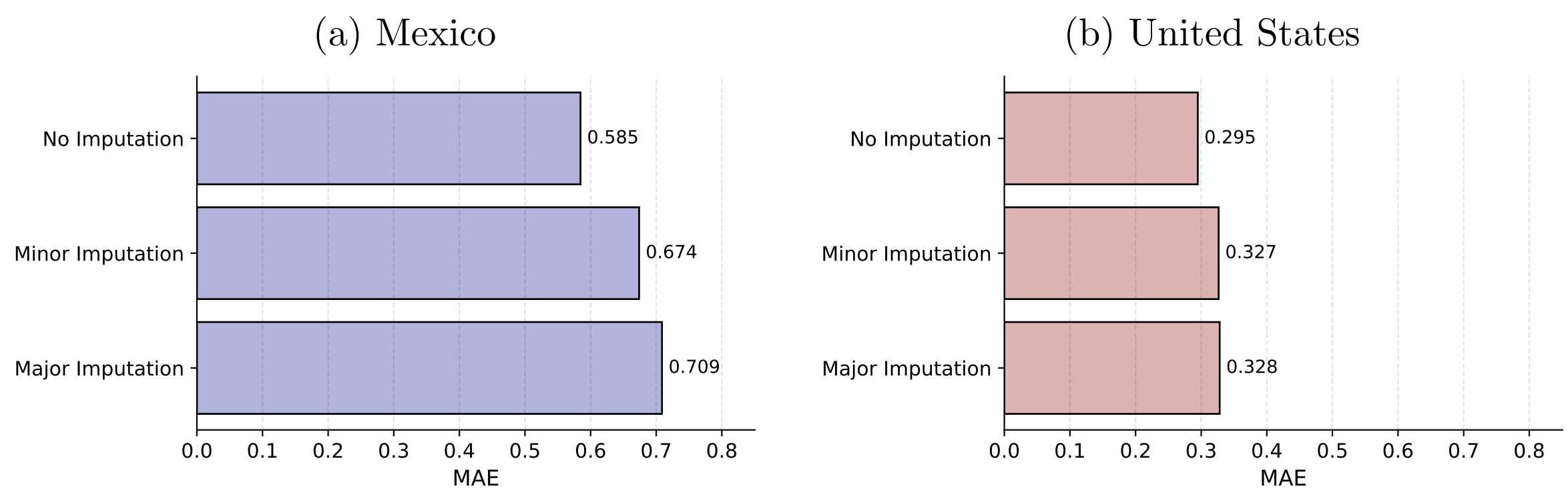

Figure A.10: Model performance by imputation level

Mean Absolute Error (MAE) for predictions of years of schooling in Mexican municipalities (blue) and US counties (red) grouped by imputation level. "No Imputation": units with at least 5 tweets (complete observed data); "Minor Imputation": units with 1-4 tweets (outlier imputation based on spatial neighbors); "Major Imputation": units with 0 tweets (full imputation of all tweet content features based on spatial neighbors). Lower MAE indicates better predictive performance.

B Bias Correction

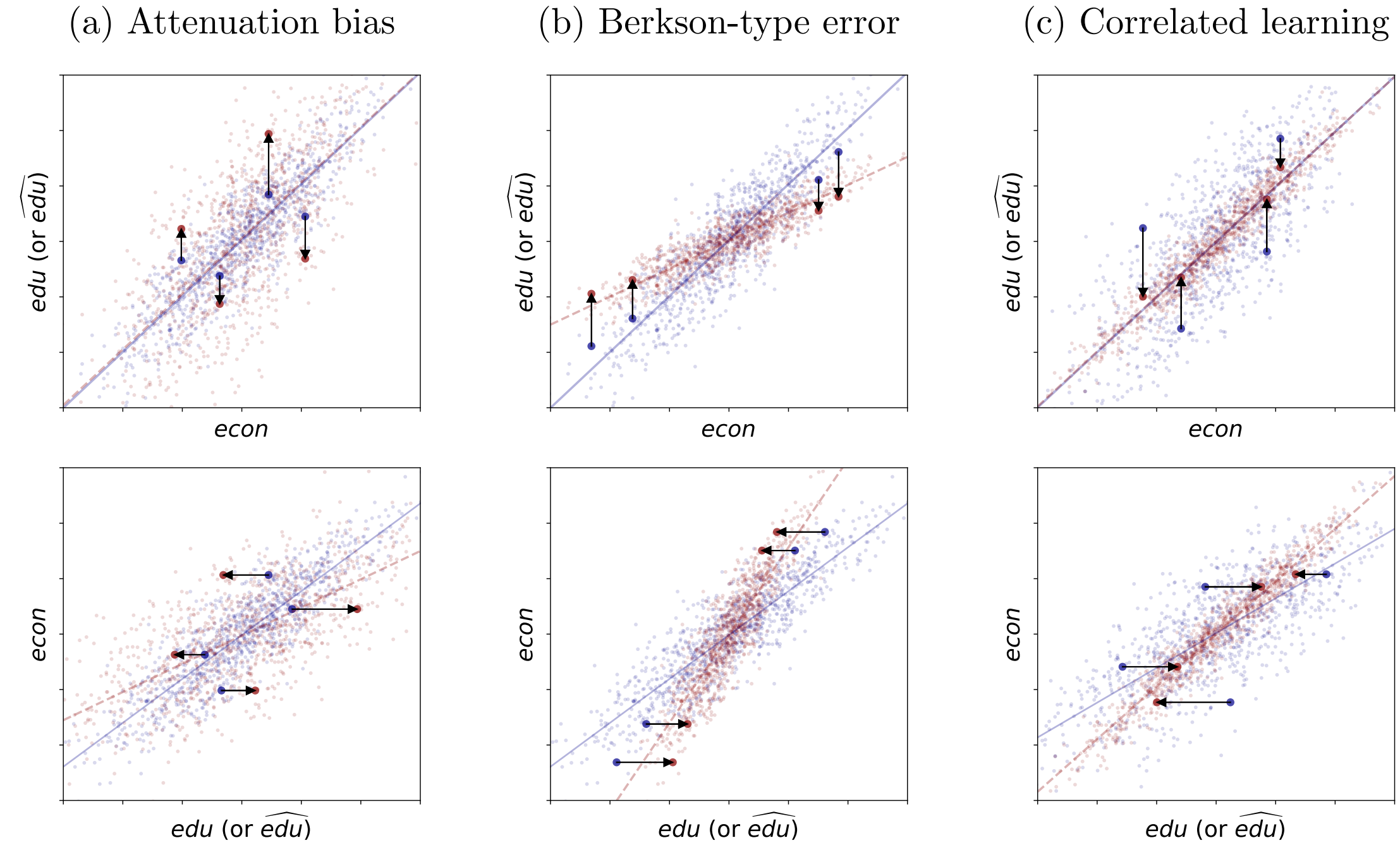

Figure B.1: Simulation of different types of biases in downstream regression tasks

Scatter plots and best linear fit for *edu* (blue) and $\widehat{edu}$ (red) with different types of measurement errors. Arrows indicate the movement of typical points as a result of each measurement error. In the upper row, *edu* (or $\widehat{edu}$) is the outcome of the regression, while it features as the explanatory variable in the lower row.

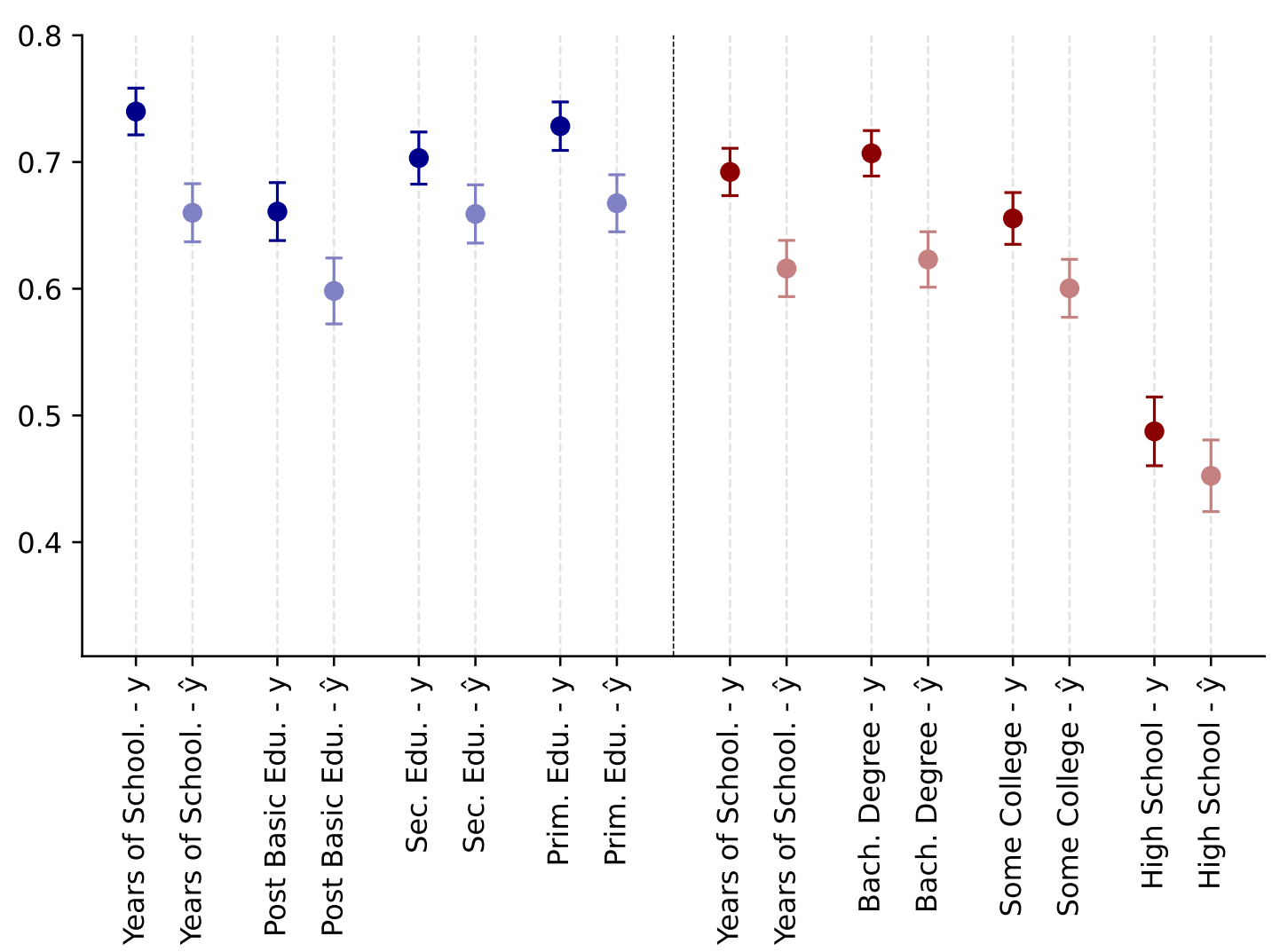

Figure B.2: Correlation of observed and predicted education with wealth or income

Correlations between true and predicted educational outcomes and wealth in Mexico (blue) as well as income in the United States (red). 95% confidence intervals shown.

C Feature Statistics

Table C.1: Survey statistics by country

Variable	Country	Mean	SD	Min	Median	Max
Years of Schooling	MX	7.83	1.49	3.40	7.72	14.55
	US	13.30	0.66	9.37	13.28	16.13
Post Basic Education	MX	0.28	0.13	0.03	0.26	0.89
Bachelor Degree	US	22.61	9.71	0.00	20.22	79.14
Secondary Education	MX	0.54	0.14	0.12	0.54	0.95
Some College	US	53.67	10.72	7.41	53.61	90.31
Primary Education	MX	0.76	0.11	0.36	0.76	0.98
High School	US	87.60	6.04	21.85	88.83	98.61
Population	MX	51,173.11	147,322.51	81.00	13,552.00	1,922,523.00
	US	105,661.95	333,146.18	57.00	25,790.00	9,829,544.00
Wealth Index	MX	0.68	0.12	0.07	0.70	0.94
Income	US	57,455.86	14,582.81	22,901.00	55,143.50	160,305.00

Table C.2: Twitter penetration and usage statistics by country

Variable	Country	Mean	SD	Min	Median	Max
Tweet count	MX	1,093.25	6,363.61	0.00	8.00	119,126.00
	US	7,195.60	42,124.22	0.00	271.00	1,472,677.00
User count	MX	50.11	269.79	0.00	2.00	5,891.00
	US	299.54	1,548.14	0.00	24.00	52,602.00
Share weekdays	MX	0.70	0.22	0.00	0.72	1.00
	US	0.70	0.15	0.00	0.71	1.00
Share workhours	MX	0.29	0.21	0.00	0.28	1.00
	US	0.31	0.15	0.00	0.31	1.00
Follower count	MX	251.37	1,210.64	0.00	111.83	36,807.50
	US	304.11	754.33	0.00	229.50	24,799.80
Following count	MX	358.47	480.14	0.00	261.71	7,603.16
	US	415.74	556.26	1.00	359.50	25,202.00
Tweet count	US	2,659.14	6,237.16	1.00	1,927.00	183,023.00
	MX	2,405.62	5,565.75	1.00	961.45	79,700.00
User mobility	MX	1.61	0.75	1.00	1.50	10.00
	US	1.76	0.80	1.00	1.71	32.00
iPhone share	US	0.62	0.23	0.00	0.67	1.00
	MX	0.28	0.27	0.00	0.25	1.00
Instagram share	US	0.21	0.24	0.00	0.12	1.00
	MX	0.14	0.23	0.00	0.06	1.00
Favorites per tweet	US	1.73	8.74	0.00	1.35	463.46
	MX	3.76	23.95	0.00	1.25	892.38
Tweets per year	US	495.19	1,703.97	0.39	325.13	52,472.55
	MX	841.93	6,183.31	0.82	260.06	210,975.91
Account age	MX	5.67	2.67	-0.03	5.88	13.56
	US	7.06	1.69	-0.02	7.10	14.65

Variable	Country	Mean	SD	Min	Median	Max
Account age	MX	5.67	2.67	-0.03	5.88	13.56
	US	7.06	1.69	-0.02	7.10	14.65
Listed count	MX	2.86	7.79	0.00	1.00	151.40
	US	12.39	30.79	0.00	6.67	711.60
Followers per following	MX	0.64	2.39	0.00	0.38	68.93
	US	0.71	1.60	0.00	0.60	61.70
Share quotes	MX	0.07	0.11	0.00	0.04	1.00
	US	0.09	0.07	0.00	0.10	1.00
Share replies	MX	0.24	0.22	0.00	0.23	1.00
	US	0.22	0.13	0.00	0.24	1.00
Share verified	MX	0.00	0.03	0.00	0.00	1.00
	US	0.01	0.03	0.00	0.00	1.00
Tweet length	MX	69.94	29.48	4.00	67.53	274.00
	US	80.86	21.74	6.00	79.01	275.00
Hashtags per tweet	MX	0.30	0.51	0.00	0.17	8.00
	US	0.38	0.52	0.00	0.28	8.00
Mentions per tweet	US	0.53	0.31	0.00	0.56	4.44
	MX	0.45	0.45	0.00	0.41	7.00
Urls per tweet	US	0.38	0.52	0.00	0.28	8.00
	MX	0.30	0.51	0.00	0.17	8.00
Emojis per tweet	MX	0.81	0.70	0.00	0.72	6.67
	US	0.55	0.54	0.00	0.50	15.00

Table C.3: Error statistics by country

Variable	Country	Mean	SD	Min	Median	Max
Error typography	MX	7.71	9.03	0.00	6.57	170.57
	US	2.55	2.47	0.00	2.18	30.61
Error grammar	MX	0.17	0.54	0.00	0.00	9.80
	US	0.55	0.82	0.00	0.43	15.87
Error confusions	MX	0.14	0.51	0.00	0.00	11.11
	US	0.10	0.26	0.00	0.04	7.44
Error casing	MX	1.29	3.49	0.00	0.18	55.56
	US	1.73	2.74	0.00	1.29	60.61
Error misc	MX	0.12	1.22	0.00	0.00	47.15
	US	0.19	0.44	0.00	0.13	14.71
Error style	US	0.43	0.92	0.00	0.30	23.81
	MX	0.02	0.24	0.00	0.00	7.19
Error repetitions style	US	0.00	0.00	0.00	0.00	0.01
	MX	0.00	0.00	0.00	0.00	0.08
Error semantics	US	0.01	0.11	0.00	0.00	4.57
	MX	0.01	0.06	0.00	0.00	1.94
Error variants	US	0.01	0.06	0.00	0.00	2.51
	MX	0.07	0.72	0.00	0.00	25.64
Error punctuation	US	1.02	1.39	0.00	0.91	32.26
	MX	0.66	1.83	0.00	0.24	35.51
Error typos	US	7.19	5.94	0.00	6.78	181.82
	MX	12.47	13.82	0.00	10.16	285.71
Error total	MX	25.28	17.43	0.00	22.87	285.71
	US	13.87	7.68	0.00	13.16	181.82

Variable	Country	Mean	SD	Min.	Median	Max.
Error expressions	MX	0.02	0.15	0.00	0.00	2.82
Error redundancy	MX	0.00	0.01	0.00	0.00	0.23
Error prepositions	MX	0.00	0.00	0.00	0.00	0.04
Error verb agreement	MX	0.02	0.27	0.00	0.00	10.42
Error misspelling	MX	1.18	4.04	0.00	0.50	125.00
Error proper nouns	MX	0.00	0.01	0.00	0.00	0.34
Error diacritics	MX	1.07	1.60	0.00	0.68	16.67
Error context	MX	0.00	0.01	0.00	0.00	0.38
Error repetitions	MX	0.01	0.14	0.00	0.00	5.38
Error norm change	MX	0.07	0.28	0.00	0.00	5.56
Error noun agreement	MX	0.26	0.80	0.00	0.02	14.93
Error collocations	US	0.01	0.20	0.00	0.00	8.20
Error nonstandard	US	0.00	0.02	0.00	0.00	0.49
Error redundancy	US	0.05	0.42	0.00	0.01	18.87
Errror compounding	US	0.02	0.13	0.00	0.00	5.32

Table C.4: Topic statistics by country

Variable	Country	Mean	SD	Min	Median	Max
Topic arts & culture	MX	3.87	2.28	0.30	3.39	24.89
	US	2.97	1.54	0.27	2.68	29.31
Topic business	MX	3.00	3.15	0.26	2.33	46.23
	US	2.79	2.10	0.24	2.55	36.69
Topic celebrity	MX	6.83	5.06	0.31	6.33	49.20
	US	6.10	2.95	0.37	6.33	42.41
Topic daily life	MX	26.03	8.28	1.22	25.48	59.21
	US	21.30	5.91	1.26	20.74	60.28
Topic family	MX	4.03	3.09	0.32	3.52	32.72
	US	3.99	1.89	0.30	3.74	33.24
Topic fashion	MX	1.33	1.47	0.20	1.00	18.35
	US	1.67	1.49	0.19	1.49	38.24
Topic films	MX	4.12	4.23	0.36	3.38	64.47
	US	4.25	3.46	0.26	4.04	67.58
Topic fitness & health	US	2.42	1.61	0.31	2.33	37.45
	MX	2.38	2.36	0.22	1.88	31.72
Topic food & dining	US	3.12	3.35	0.16	2.72	48.61
	MX	2.29	3.29	0.15	1.42	47.21
Topic gaming	MX	1.47	1.55	0.17	1.14	31.18
	US	2.24	1.31	0.29	2.07	19.91

Variable	Country	Mean	SD	Min	Median	Max
Topic educational	MX	1.45	1.65	0.15	1.10	24.93
	US	1.80	1.65	0.16	1.52	30.81
Topic music	MX	4.67	5.57	0.13	3.74	69.11
	US	4.01	3.14	0.12	3.85	66.19
Topic news	MX	13.00	9.50	0.34	11.69	83.39
	US	12.29	5.85	0.40	12.00	80.56
Topic hobbies	MX	7.31	3.21	0.49	6.92	34.64
	US	5.12	2.06	0.34	4.82	26.46
Topic relationships	MX	6.27	3.70	0.29	5.78	27.32
	US	4.76	1.98	0.30	4.58	21.19
Topic science	MX	1.87	2.79	0.23	1.22	54.54
	US	1.69	1.71	0.29	1.49	46.50
Topic sports	MX	5.87	6.44	0.31	4.54	65.84
	US	15.14	9.31	0.12	14.36	85.78
Topic travel	MX	3.28	3.37	0.24	2.31	30.65
	US	2.95	2.37	0.23	2.19	30.52
Topic youth	MX	0.93	1.26	0.18	0.68	22.34
	US	1.40	1.34	0.18	1.15	23.65

Table C.5: Sentiment statistics by country

Variable	Country	Mean	SD	Min	Median	Max
Sentiment negative	MX	0.16	0.12	0.00	0.16	0.95
	US	0.16	0.08	0.00	0.17	0.91
Sentiment positive	MX	0.38	0.18	0.01	0.37	0.99
	US	0.50	0.13	0.01	0.48	0.99
Hate speech	MX	0.04	0.03	0.01	0.04	0.42
	US	0.05	0.02	0.01	0.04	0.33
Offensive language	MX	0.15	0.07	0.03	0.15	0.89
	US	0.16	0.06	0.03	0.16	0.83

Table C.6: Network statistics by country

Variable	Country	Mean	SD	Min	Median	Max
Network in degree	MX	0.14	0.87	0.00	0.00	15.17
	US	0.36	2.17	0.00	0.01	67.43
Network out degree	MX	0.14	0.79	0.00	0.00	14.65
	US	0.36	1.91	0.00	0.01	56.15
Network clos. centr.	MX	0.16	0.18	0.00	0.00	0.55
	US	0.34	0.16	0.00	0.40	0.68
Network pagerank	MX	0.00	0.00	0.00	0.00	0.06
	US	0.00	0.00	0.00	0.00	0.05

D Feature Descriptions

This appendix describes all features included in our analysis. Variables marked as "log scale" in the tables below are log-transformed before being used in the model. Following Stahel (2000), we use $\log(x + c)$ to deal with zeros, with x as the values of a particular feature and $c = Q_{0.25}^2 \,/\, Q_{0.75}$, where $Q_{0.25}$ and $Q_{0.75}$ are the first and third quartiles based on feature values $x > 0$.

Table D.1: Survey indicator description

Label	Description
Years of Schooling	Average years of schooling in municipality (MX) or county (US) according to census. We approximate years of schooling for the US by attainment statistics (see main text)
Post Basic Education	Share of population with post basic education
Secondary Education	Share of population with secondary education
Primary Education	Share of population with primary education
Wealth Index	Index based on share of households that have 13 wealth related items according to the Mexican census, sum across standardized items
Bachelor Degree	Share of county level population with some college level education
Some College	Share of population with a bachelor degree
High School	Share of population with high school education
Income	Income statistics provided by US census
Population	Population counts according to census

Table D.2: Network indicator description

Label	Description
Network in degree	Number outgoing references measured by mentions and quotes (log scale)
Network out degree	Number incoming references measured by mentions and quotes (log scale)
Network clos. centr.	Pagerank for municipalities (MX) or counties (US) according to respective network based on mentions and quotes (log scale)
Network pagerank	Closeness centrality for municipalities (MX) or counties (US) according to respective network based on mentions and quotes (log scale)

Table D.3: Twitter penetration and usage indicator description

Label	Description
Tweet count	Number of tweets
User count	Number of users
Share weekdays	Share of tweets created during weekdays (Monday-Friday)
Share workhours	Share of tweets created during workhours (Monday-Friday, 8:00am-4:00pm))
Follower count	Median number of followers per user (log scale)
Following count	Median number of friends per user (log scale)
Tweet count	Median number of tweets per user (log scale)
User mobility	Average number of municipalities (MX) or counties (US) users tweet from (log scale)
iPhone share	Share of tweets sent from an iPhone
Instagram share	Share of tweets sent via Instagram (log scale)
Favorites per tweet	Number of likes per tweet, median (log scale)
Tweets per year	Median number of tweets per year (log scale)
Account age	Age of average account

Table D.4: Twitter penetration and usage indicator description

Label	Description
Account age	Age of average account
Listed count	Average number of public lists user is a member of (log scale)
Followers per following	Number of followers divided by number of accounts a user follows, median (log scale)
Share quotes	Share of tweets that are quotes (log scale)
Share replies	Share of tweets that are replies (log scale)
Share verified	Share of verified users (log scale)
Tweet length	Average number of characters per tweet (log scale)
Hashtags per tweet	Average number of hashtags per tweet (log scale)
Mentions per tweet	Average number of mentions per tweet (log scale)
Urls per tweet	Average number of urls per tweet (log scale)
Emojis per tweet	Number of emoji per tweet (log scale)

Table D.5: Error indicator description (both countries)

Label	Description
Error total	Number of errors per character (log scale)
Error casing	Casing error (log scale)
Error confusions	Word confusions (log scale)
Error grammar	Grammar error (log scale)
Error variants	Errors regarding American and British English (log scale)
Error misc	Miscellaneous error (log scale)
Error punctuation	Punctuation error (log scale)
Error repetitions style	Style error related to repetitions (log scale)
Error semantics	Semantic error (log scale)
Error style	Style error (log scale)
Error typography	Typography error (log scale)
Error typos	Typo (log scale)

Table D.6: Error indicator description (country-specific)

Label	Description
Error noun agreement	Noun verb agreement error (log scale)
Error verb agreement	Verb subject agreement error (log scale)
Error norm change	Deviation from linguistic norms (log scale)
Error collocations	Collocation error (log scale)
Error compounding	Compounding error (log scale)
Error context	Context dependent error (log scale)
Error diacritics	Errors regarding accents (diacritic marks, log scale)
Error expressions	Incorrect expression (log scale)
Error misspelling	Misspelling (log scale)
Error nonstandard	Error related to non-standard English (log scale)
Error prepositions	Error related to prepositions (log scale)
Error proper nouns	Error related to proper nouns (log scale)
Error redundancy	Redundancy in text (log scale)
Error redundancy	Redundancy in text (log scale)
Error repetitions	Repetition in text (log scale)

Table D.7: Topic indicator description

Label	Description
Topic arts & culture	Share of tweets classified into the arts & culture topic (log scale)
Topic business	Share of tweets classified into the business & entrepreneurs topic (log scale)
Topic celebrity	Share of tweets classified into the celebrity & pop culture topic (log scale)
Topic daily life	Share of tweets classified into the diaries & daily life topic (log scale)
Topic family	Share of tweets classified into the family topic (log scale)
Topic fashion	Share of tweets classified into the fashion & style topic (log scale)
Topic films	Share of tweets classified into the films, tv & video topic (log scale)
Topic fitness & health	Share of tweets classified into the fitness & health topic (log scale)
Topic food & dining	Share of tweets classified into the food & dining topic (log scale)
Topic gaming	Share of tweets classified into the gaming topic (log scale)

Table D.8: Topic indicator description

Label	Description
Topic educational	Share of tweets classified into the learning & educational topic (log scale)
Topic music	Share of tweets classified into the music topic (log scale)
Topic news	Share of tweets classified into the news & social concern topic (log scale)
Topic hobbies	Share of tweets classified into the other hobbies topic (log scale)
Topic relationships	Share of tweets classified into the relationships topic (log scale)
Topic science	Share of tweets classified into the science & technology topic (log scale)
Topic sports	Share of tweets classified into the sports topic (log scale)
Topic travel	Share of tweets classified into the travel & adventure topic (log scale)
Topic youth	Share of tweets classified into the youth & student life topic (log scale)

Table D.9: Sentiment indicator description

Label	Description
Sentiment negative	Average share of tweets with negative sentiment in contrast to positive and neutral
Sentiment positive	Average share of tweets with positive sentiment in contrast to negative and neutral
Hate speech	Score indicating hate speech, average (log scale)
Offensive language	Score indicating offensive language, average (log scale)

E Data Collection

This section expands on the data collection procedures described in Sections 2.1 and 2.2

E.1 Twitter Data

Our Twitter data was collected via the Twitter Streaming API between July and August 2021. Until early 2023, this API provided free real-time access to a 1% sample of all tweets, including the text of each tweet and a set of tweet and user characteristics. In February 2023, the free Streaming API was replaced with a paid service. Twitter described its 1% sample as random, though the exact sampling mechanism was proprietary, not fully transparent, and shown to exhibit systematic biases (Morstatter et al., 2013). Importantly, the generalizability of our approach does not require the sample to be representative of all Twitter users but only consistent across countries and time. While Twitter provided a single global API endpoint, the proprietary nature of the sampling mechanism means that regional differences in coverage cannot be entirely ruled out. However, Morstatter et al. (2013) found that when using geographic bounding boxes as collection parameters — as we do — the Streaming API captured approximately 90% of geotagged tweets rather than the nominal 1% sampling rate, leaving limited room for region-specific variation in sampling coverage.

To restrict our sample to content created by human users, we include only tweets posted through the four main channels for individual users: iPhone, Android, iPad, and Instagram. For tweets posted through Instagram, we additionally exclude those that use the default text ("Just posted a photo @...") rather than a message specified by the user. Tweets posted through the Twitter website are not included because they do not have associated coordinate information. These filters exclude tweets generated through third-party APIs from platforms such as Foursquare or CareerArc, corresponding to approximately 1 percent of geolocated tweets in Mexico and 7 percent in the United States.

We assign each tweet to a single municipality (Mexico) or county (United States) based on its coordinate information. For tweets with exact coordinates, this assignment is straightforward. For tweets with bounding box coordinates at the city, point-of-interest, or neighborhood level, assignment to the geographical unit containing the bounding box centroid yields correct results in most cases. However, in the Mexican case, where the location precision for tweets tends to be lower and where Twitter's "city-level" precision refers to municipalities rather than to places within municipalities, we combine spatial joins with name matching to ensure that all tweets are assigned to the correct entity.

E.2 Survey Data

Our ground-truth education data comes from the 2020 Mexican census, publicly available at `https://www.inegi.org.mx/datosabiertos/`, and from the American Community Survey 2017–2021 5-year estimates, accessible at `https://www.ers.usda.gov/data-products/county-level-data-sets/`. The Mexican census reports average years of schooling directly, while the American Community Survey reports the share of the

population holding different educational degrees. Following Barro and J. W. Lee (2013), we approximate county-level years of schooling for the United States as

$$\sum_j h_j \, Dur_j, \tag{1}$$

where h_j indicates the proportion of the population that has attained education level j and Dur_j indicates the corresponding duration to attain that level. Estimates for Dur_j are derived from the *2021 Annual Social and Economic (ASEC) Supplement* of the *Current Population Survey.*

The reference populations differ slightly across outcome variables and countries. For Mexico, years of schooling and primary and secondary completion are reported for the population aged 16 or older, while the share with post-basic education is defined for adults aged 18 or older. For the United States, all education statistics from the American Community Survey refer to the population aged 25 or older.

We further obtain population counts from the same sources, allowing us to compute population, tweet, and user densities. Because population data is widely available across countries, its inclusion does not limit the external validity of our approach.

References

Akrami, Haleh, Anand A Joshi, Sergül Aydöre, and Richard M Leahy (2022). "Deep quantile regression for uncertainty estimation in unsupervised and supervised lesion detection". *The Journal of Machine Learning for Biomedical Imaging* 1, 008.

Angelopoulos, Anastasios N and Stephen Bates (2021). "A gentle introduction to conformal prediction and distribution-free uncertainty quantification". *arXiv preprint arXiv:2107.07511*.

Barro, Robert J. and Jong Wha Lee (2013). "A new data set of educational attainment in the world, 1950–2010". *Journal of Development Economics* 104, 184–198.

Lundberg, Scott M and Su-In Lee (2017). "A Unified Approach to Interpreting Model Predictions". *Advances in Neural Information Processing Systems 30*. Ed. by I. Guyon, U. V. Luxburg, S. Bengio, H. Wallach, R. Fergus, S. Vishwanathan, and R. Garnett. Curran Associates, Inc., 4765–4774.

Maddox, Wesley J, Pavel Izmailov, Timur Garipov, Dmitry P Vetrov, and Andrew Gordon Wilson (2019). "A simple baseline for bayesian uncertainty in deep learning". *Advances in Neural Information Processing Systems* 32.

Morstatter, Fred, Jürgen Pfeffer, Huan Liu, and Kathleen Carley (2013). "Is the sample good enough? Comparing data from Twitter's streaming API with Twitter's firehose". *Proceedings of the International AAAI Conference on Web and Social Media*. Vol. 7. 1, 400–408.

Stahel, Werner A. (2000). *Statistische Datenanalyse: Eine Einfuehrung fuer Naturwissenschaftler*. Vieweg+Teubner Verlag Wiesbaden.
